# In Vivo Measurement of Cell-Type-Specific Synaptic Connectivity and Synaptic Transmission in Layer 2/3 Mouse Barrel Cortex

**DOI:** 10.1016/j.neuron.2014.11.025

**Published:** 2015-01-07

**Authors:** Aurélie Pala, Carl C.H. Petersen

**Affiliations:** 1 Laboratory of Sensory Processing, Brain Mind Institute, Faculty of Life Sciences, École Polytechnique Fédérale de Lausanne (EPFL), Lausanne 1015, Switzerland

## Abstract

Intracellular recordings of membrane potential in vitro have defined fundamental properties of synaptic communication. Much less is known about the properties of synaptic connectivity and synaptic transmission in vivo. Here, we combined single-cell optogenetics with whole-cell recordings to investigate glutamatergic synaptic transmission in vivo from single identified excitatory neurons onto two genetically defined subtypes of inhibitory GABAergic neurons in layer 2/3 mouse barrel cortex. We found that parvalbumin-expressing (PV) GABAergic neurons received unitary glutamatergic synaptic input with higher probability than somatostatin-expressing (Sst) GABAergic neurons. Unitary excitatory postsynaptic potentials onto PV neurons were also faster and more reliable than inputs onto Sst neurons. Excitatory synapses targeting Sst neurons displayed strong short-term facilitation, while those targeting PV neurons showed little short-term dynamics. Our results largely agree with in vitro measurements. We therefore demonstrate the technical feasibility of assessing functional cell-type-specific synaptic connectivity in vivo, allowing future investigations into context-dependent modulation of synaptic transmission.

## Highlights

• Single-cell optogenetics for precise stimulation of action potentials in vivo
• In vivo whole-cell recordings from genetically defined postsynaptic GABAergic neurons
• Parvalbumin-expressing neurons receive strong, fast, and reliable excitatory input
• Somatostatin-expressing neurons receive longer-lasting, facilitating excitatory input

## Teaser

Pala and Petersen use single-cell optogenetics and two-photon targeted whole-cell recordings to measure synaptic connectivity and synaptic transmission in vivo from excitatory neurons onto parvalbumin-expressing and somatostatin-expressing GABAergic neurons in layer 2/3 of mouse barrel cortex.

## Introduction

Chemical synaptic transmission is fundamental to brain function and forms the major mechanism for rapid signaling between neurons. Action potentials (APs) evoke calcium influx, driving exocytosis of synaptic vesicles. Fast postsynaptic potentials are evoked by the released neurotransmitter acting upon ionotropic receptors. Early investigations of synaptic transmission in vitro at the frog neuromuscular junction revealed quantal postsynaptic potentials corresponding to release of single synaptic vesicles (Del Castillo and Katz, 1954). The development of in vitro brain slice preparations together with multiple simultaneous intracellular electrophysiological recordings allowed the functional properties of glutamatergic synaptic connectivity and synaptic transmission to be studied in detail between identified pre- and postsynaptic neurons of the mammalian neocortex (Buhl et al., 1997; Reyes et al., 1998; Galarreta and Hestrin, 1998; Beierlein et al., 2003; Holmgren et al., 2003; Koester and Johnston, 2005; Lefort et al., 2009; Hofer et al., 2011; Avermann et al., 2012). These in vitro measurements revealed cell-type-specific synaptic connectivity and cell-type-specific properties of synaptic transmission. Since glutamatergic synapses provide the major excitatory drive for neocortical circuits, these in vitro measurements of glutamatergic synaptic connectivity and synaptic transmission are of fundamental importance for understanding network function. However, due to differences in concentrations of ions, neurotransmitters, neuromodulators, and other molecules, synaptic transmission might be different in vivo. In addition, synaptic connectivity might differ since axonal and dendritic arborisations are truncated by slicing procedures for in vitro recordings. It is therefore of fundamental importance to measure synaptic connectivity and synaptic transmission in vivo.

Few studies have directly investigated synaptic transmission between identified neocortical neurons in vivo, presumably due to the technical difficulties in obtaining intracellular recordings from connected pairs of neurons in vivo (Matsumura et al., 1996; Crochet et al., 2005; Bruno and Sakmann, 2006; Yu and Ferster, 2013). Moreover, it is unknown how synaptic transmission differs among specific neocortical cell types in vivo. Here, we develop a robust technical approach for measuring synaptic transmission between identified neurons in vivo and apply it to investigate excitatory synaptic transmission between single identified layer 2/3 (L2/3) excitatory neurons and two different types of genetically defined postsynaptic GABAergic neurons.

## Results

**Figure 1:**
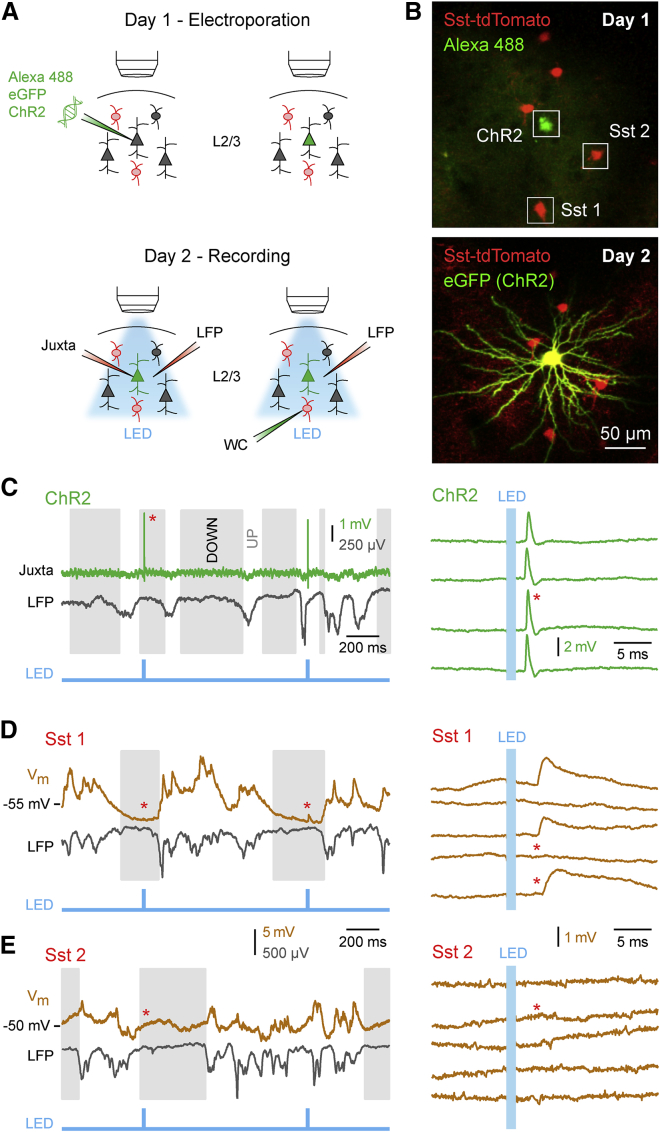
In Vivo Measurement of uEPSPs (A) On day 1, eGFP- and ChR2-encoding plasmid DNAs together with Alexa 488 dye are electroporated into a single excitatory neuron in L2/3 mouse barrel cortex. On day 2, juxtacellular recording of the ChR2-expressing excitatory neuron is carried out to assess optogenetic control of AP firing. Whole-cell (WC) recordings of nearby tdTomato-expressing neurons are then performed sequentially to measure synaptic potentials. Local field potential (LFP) is recorded simultaneously. (B) Example in vivo two-photon images of a single L2/3 excitatory neuron filled with Alexa 488 dye in a Sst-Cre × LSL-tdTomato mouse taken immediately after electroporation (above) and 24 hr later showing eGFP expression in soma and dendrites (below). (C) Juxtacellular recording of the AP firing response to a single 1 ms light pulse delivered at 1 Hz to the ChR2-expressing neuron in (B). LFP recording allowed identification of DOWN (gray) and UP states (white) (left). A single AP was elicited with precise timing by each light pulse during DOWN states (right). (D) Whole-cell recording of a synaptically connected neuron, Sst 1 in (B), with simultaneous LFP recording (left). Example single-trial uEPSPs and synaptic failures recorded during DOWN states (right). (E) Same as (D), but for an unconnected Sst neuron, Sst 2 in (B). See also Movie S1.

To investigate excitatory synaptic transmission in vivo, we combined optogenetic control of a single excitatory presynaptic neuron with simultaneous whole-cell membrane potential (V_m_) recordings to measure unitary excitatory postsynaptic potentials (uEPSPs) in identified GABAergic neurons in L2/3 barrel cortex of the anesthetized mouse (Figure 1A). We delivered plasmid DNA encoding a fast variant of channelrhodopsin-2 (ChR2) (Berndt et al., 2011) and eGFP to a single L2/3 neuron using two-photon guided electroporation (Movie S1, available online) (Kitamura et al., 2008). After 1 day, eGFP expression level was sufficiently high to allow morphological validation of the excitatory nature of the electroporated neuron (Figure 1B). In every experiment, we first measured the reliability and temporal precision of the optogenetically evoked presynaptic APs through targeted juxtacellular recording of the ChR2-expressing neuron (Figure 1C). Simultaneous recording of the local field potential (LFP) allowed us to distinguish periods of neuronal network quiescence (DOWN states) from periods of spontaneous depolarization and activity (UP states) (Steriade et al., 1993; Cowan and Wilson, 1994). We then recorded the V_m_ response to optogenetic single-cell stimulation in genetically defined GABAergic neurons expressing the fluorescent protein tdTomato. In some postsynaptic V_m_ recordings we observed optogenetically evoked uEPSPs, defining a synaptically connected pair of neurons (Figure 1D). On the other hand, no uEPSPs were detected in V_m_ recordings from other cells, defining unconnected pairs of neurons (Figure 1E).

### Reliable and Precise Optogenetic Control of Action Potential Firing

Quantification of synaptic connectivity and the properties of uEPSPs requires reliable and precise generation of single APs in single identified presynaptic neurons. We therefore measured the reliability and temporal precision of the APs evoked in single ChR2-expressing neurons by optogenetic stimulation in vivo.

**Figure 2:**
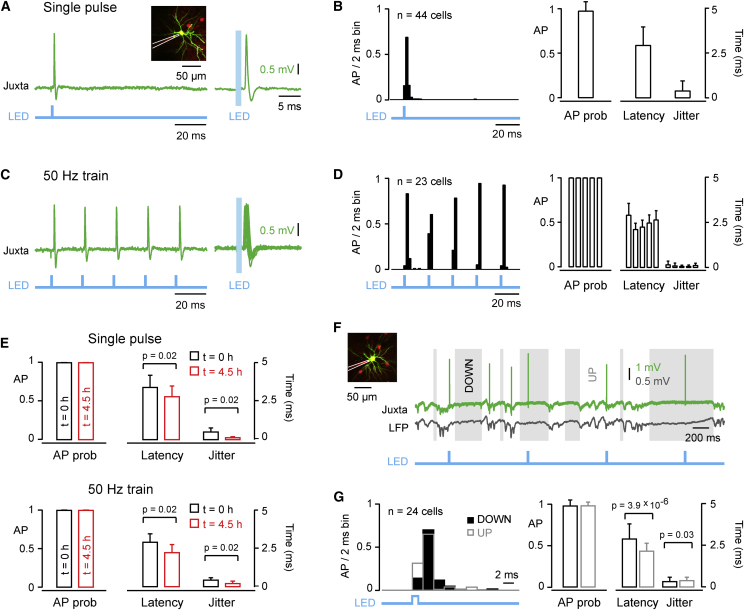
Precise Optogenetic Stimulation of Action Potential Firing in Single Excitatory Neurons In Vivo (A) Example single AP elicited by a single 1 ms light pulse recorded juxtacellularly in a L2/3 ChR2-expressing excitatory neuron. (B) Population peristimulus time histogram of light-evoked AP timing (left) and light-evoked AP probability, latency, and jitter (right) for single 1 ms light pulses delivered during the DOWN states. (C) Same cell as in (A), but for an optogenetic stimulus made of a 50 Hz train of five 1 ms light pulses. (D) Same analysis as in (B), but for an optogenetic stimulus made of a 50 Hz train of five 1 ms light pulses. (E) Light-evoked AP probability, latency, and jitter quantified at the beginning (black, t = 0 hr) and end (red, t = 4.5 hr) of the recording session for single 1 ms light pulses (above) and 50 Hz trains of five 1 ms light pulses (below). (F) Example APs elicited by a single 1 ms light pulse delivered at 1 Hz recorded juxtacellularly during UP and DOWN states. (G) Population peristimulus time histogram of light-evoked AP timing (left) and light-evoked AP probability, latency, and jitter (right) for 1 ms optogenetic stimuli occurring in DOWN (black) and UP states (gray). Data are represented as mean ± SD. Two-tail Wilcoxon signed-rank test assessed statistical significance. See also Figure S1.

We first analyzed APs evoked during the hyperpolarized quiescent DOWN state of the neocortex (Figures 2A and 2B). We delivered single 1 ms blue light flashes at 1 Hz and found that single APs could be evoked reliably (98% ± 10%, n = 44) with a short latency (2.9 ± 1.0 ms) and a low jitter (0.4 ± 0.5 ms) relative to the onset of the blue light flash. We next examined the ability of the optogenetic stimulus to drive high-frequency trains of APs. Using the same light intensity used for evoking single APs, we delivered trains of five 1 ms blue light flashes at 20 Hz (Figure S1) and 50 Hz (Figures 2C and 2D). At these high frequencies, APs could be elicited with equally high probability (20 Hz 100% ± 0%, n = 17; 50 Hz 100% ± 0%, n = 23), short latency (20 Hz 3.2 ± 0.7 ms; 50 Hz 2.5 ± 0.4 ms), and low jitter (20 Hz 0.2 ± 0.1 ms; 50 Hz 0.3 ± 0.1 ms).

Our recording sessions typically lasted ∼4.5 hr, and it was therefore important to test the stability of the optogenetic stimulation over long time scales. In a subset of experiments (n = 7), we recorded the APs elicited in the ChR2-expressing neuron at both the beginning and the end of the recording session, delivering the same light stimuli in both cases (Figure 2E). Over this time period, we found that the high probability of evoking APs in response to a single light flash was unchanged (0 hr, 100% ± 0%; 4.5 hr, 100% ± 0%; p = 1), while AP latency (0 hr, 3.4 ± 0.8 ms; 4.5 hr, 2.8 ± 0.7 ms; p = 0.02) and jitter (0 hr, 0.5 ± 0.3 ms; 4.5 hr, 0.2 ± 0.0 ms; p = 0.02) decreased. Similarly, high-frequency optogenetic stimulation was stable in terms of AP probability but also showed shorter AP latency and reduced jitter over ∼4.5 hr, which could result from gradually increasing expression levels of ChR2 over the duration of the experiment.

In addition, we examined the impact of spontaneous activity upon the reliability and timing of optogenetically evoked APs (Figures 2F and 2G). We found an equally high light-evoked AP probability in UP states (99% ± 4%) compared to DOWN states (99% ± 7%, p = 1, n = 24), with a slightly higher AP jitter (UP 0.43 ± 0.19 ms; DOWN 0.37 ± 0.25 ms; p = 0.03) and shorter latency (UP 2.2 ± 0.5 ms; DOWN 2.9 ± 0.9 ms; p = 3.9 × 10^−6^) in UP states compared to DOWN states (Mateo et al., 2011).

In summary, single-cell electroporation of a fast variant of ChR2 allows precise and reliable APs to be optogenetically evoked in L2/3 pyramidal neurons by 1 ms blue light flashes at 1 Hz, 20 Hz, and 50 Hz over many hours during periods of both spontaneous network quiescence and activity, therefore making single-cell optogenetic stimulation well suited for studying uEPSPs.

### Unitary Excitatory Synaptic Inputs onto Parvalbumin- and Somatostatin-Expressing GABAergic Neurons

Using two-photon microscopy we targeted whole-cell recordings to parvalbumin-expressing (PV) GABAergic neurons (n = 45; identified in PV-Cre × LSL-tdTomato mice) and somatostatin-expressing (Sst) GABAergic neurons (n = 59; identified in Sst-Cre × LSL-tdTomato mice) (Figure S2). Input resistance (PV 47 ± 22 MΩ; Sst 203 ± 45 MΩ; p = 1.9 × 10^−16^) and membrane time constant (Tau) (PV 3.6 ± 2.5 ms; Sst 17.7 ± 6.4 ms; p = 1.1 × 10^−14^) were larger in Sst compared to PV neurons (Figure S3 and Table S1). AP half-width was smaller in PV than Sst neurons, but AP threshold was similar in both cell types (Figure S3 and Table S1). Mean V_m_ was more depolarized in Sst compared to PV neurons (PV −66.1 ± 6.0 mV; Sst −59.9 ± 5.4 mV; p = 3.4 × 10^−8^), while the spontaneous AP rate of PV neurons was higher than that of Sst neurons (PV 5.1 ± 4.1 Hz; Sst 1.0 ± 1.6 Hz; p = 6.2 × 10^−10^) (Figure S3 and Table S1). The amplitude of slow (1–5 Hz) V_m_ fluctuations was smaller in Sst neurons compared to PV neurons, and slow V_m_ oscillations were highly correlated to the local field potential (LFP) for PV neurons but less correlated for Sst neurons (Figure S3 and Table S1). These two types of GABAergic neurons therefore have diverse intrinsic electrophysiological features in vivo, and their distinct patterns of spontaneous membrane potential fluctuations might be driven by different synaptic input.

**Figure 3:**
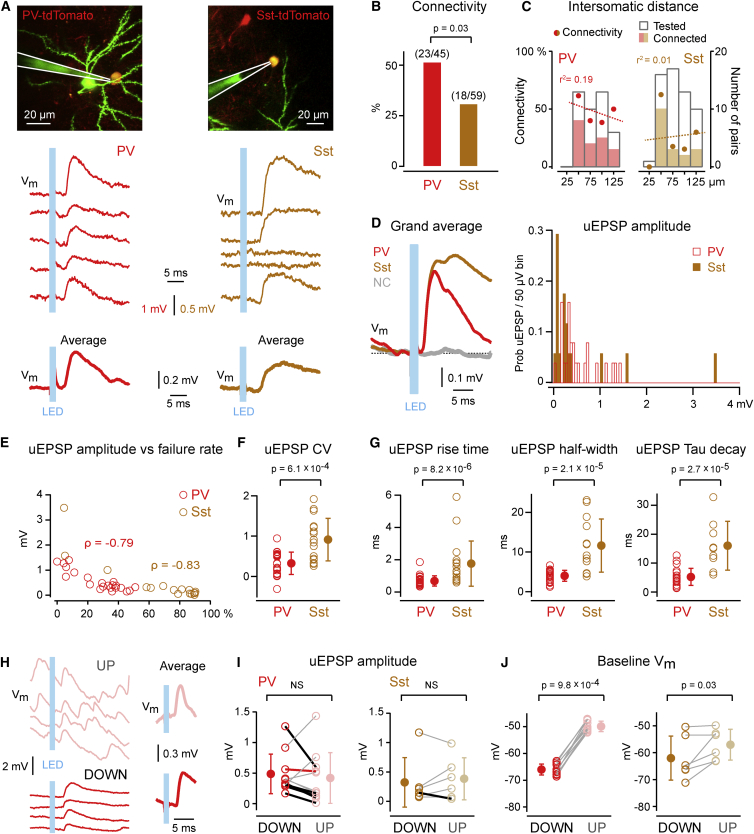
Cell-Type-Specific Features of Excitatory Synaptic Transmission In Vivo (A) Example whole-cell recording of uEPSPs elicited in a PV neuron (red) and a Sst neuron (brown) during DOWN states by 1 ms light pulses. Single trial uEPSPs are shown above and average uEPSP below. The in vivo two-photon images show the whole-cell recording pipette (Alexa 488 dye, green), the recorded tdTomato-expressing neuron (yellow), and part of the presynaptic eGFP- and ChR2-expressing neuron (green). (B) Connectivity rate is higher from excitatory neurons onto PV neurons than onto Sst neurons. (C) Connectivity rate is uncorrelated with intersomatic distance for both Exc→PV (p = 0.56) (left) and Exc→Sst pairs (p = 0.89) (right) over the short range tested. (D) uEPSP grand average of all connected PV and Sst neurons, as well as that of all nonconnected (NC) neurons (gray) (left) and uEPSP amplitude distribution (right). The uEPSP amplitude for each cell was computed as the average across both failure and success trials. (E) uEPSP amplitude is anticorrelated with the failure rate for both Exc→PV and Exc→Sst synapses. (F) uEPSP coefficient of variation (CV) is larger for Sst neurons compared to PV neurons. (G) uEPSP 20%–80% rise time, full-width at half-maximum amplitude, and exponential decay time constant (Tau) are slower for Sst neurons compared to PV neurons. (H) Example whole-cell recording of uEPSPs elicited in a PV neuron during DOWN (below) and UP states (above) by 1 ms light pulses. Single trial uEPSPs are shown on the left and average uEPSPs on the right. (I) uEPSPs elicited in DOWN states on average have an amplitude similar to that of uEPSPs elicited in UP states for both PV and Sst neurons (left). One Sst and five PV neurons show a significant decrease in uEPSP amplitude in UP compared to DOWN states (black lines). Red line represents neuron in (H). (J) Baseline V_m_ at uEPSP onset is more depolarized in UP compared to DOWN states for both PV and Sst neurons (right). Data are represented as mean ± SD. χ^2^ test assessed for statistical difference in connectivity rates. Linear regression tested distance dependence of connectivity. Two-tail Wilcoxon rank-sum test assessed the difference in uEPSP CV, rise time, half-width, and Tau decay. Two-tail Wilcoxon signed-rank test assessed the differences in uEPSP amplitude and baseline V_m_ between UP and DOWN states. Spearman’s ρ assessed the correlation between uEPSP amplitude and failure rate. See also Figures S2 and S3 and Tables S1 and S2.

By optogenetically stimulating the presynaptic ChR2-expressing excitatory neuron, we assessed the excitatory synaptic connectivity onto these two types of GABAergic neurons during the DOWN state (Figure 3A). The connection probability between excitatory and PV neurons (51%; connected/tested, 23/45) was significantly higher (p = 0.03) than the connection probability between excitatory and Sst neurons (31%; connected/tested, 18/59) (Figure 3B). Within the small range of distances explored (<125 μm), we did not find a correlation of the synaptic connectivity with respect to the distance separating the somata of the presynaptic and the postsynaptic neurons (PV r^2^ = 0.19, p = 0.56; Sst r^2^ = 0.01, p = 0.89) (Figure 3C).

The distribution of uEPSP amplitudes during the DOWN state in PV and Sst neurons was different (PV median 0.39 mV; Sst median 0.21 mV; p = 0.03), although means were similar (PV 0.53 ± 0.39 mV; Sst 0.50 ± 0.86 mV (Figure 3D and Table S2). The failure rate of synaptic transmission was lower in PV neurons compared to Sst neurons (PV 27% ± 16%; Sst 68% ± 30%; p = 0.0001) and inversely related to uEPSP amplitude in both neuron types (PV ρ = −0.79, p = 2.1 × 10^−5^; Sst ρ = −0.83, p = 6.6 × 10^−5^) (Figure 3E and Table S2). Similarly, the coefficient of variation of uEPSP amplitude was smaller in PV neurons compared to Sst neurons (PV 0.33 ± 0.28; Sst 0.92 ± 0.53; p = 6.1 × 10^−4^) (Figure 3F and Table S2).

The time course of uEPSPs also differed strongly between PV and Sst neurons. The 20%–80% rise time of uEPSPs was faster in PV than in Sst neurons (PV 0.68 ± 0.32 ms; Sst 1.76 ± 1.40 ms; p = 8.2 × 10^−6^). The half-width duration of uEPSPs was shorter in PV than Sst neurons (PV 4.0 ± 1.4 ms; Sst 11.6 ± 6.7 ms; p = 2.1 × 10^−5^), as was the exponential time constant of the decaying phase of the uEPSPs (PV 5.2 ± 3.0 ms; Sst 16.0 ± 8.5 ms; p = 2.7 × 10^−5^) (Figure 3G and Table S2).

Finally, we compared uEPSPs evoked during UP and DOWN states (Figure 3H). Although there were significant decreases in uEPSP amplitude in 5 out of 11 PV neurons and 1 out of 6 Sst neurons during UP states, overall we found that uEPSP amplitude was similar across states in both PV neurons (UP 0.41± 0.42 mV; DOWN 0.48 ± 0.33 mV; p = 0.32, n = 11) and Sst neurons (UP 0.38 ± 0.36 mV; DOWN 0.32 ± 0.42 mV; p = 0.56, n = 6) (Figure 3I). Baseline V_m_ at uEPSP onset was different between the two network states in both PV (UP −49.9 ± 1.9 mV; DOWN −66.0 ± 2.1 mV; p = 9.8 × 10^−4^) and Sst neurons (UP −57.0 ± 5.7 mV; DOWN −62.0 ± 8.2 mV; p = 0.03) (Figure 3J).

### Short-Term Synaptic Dynamics

**Figure 4:**
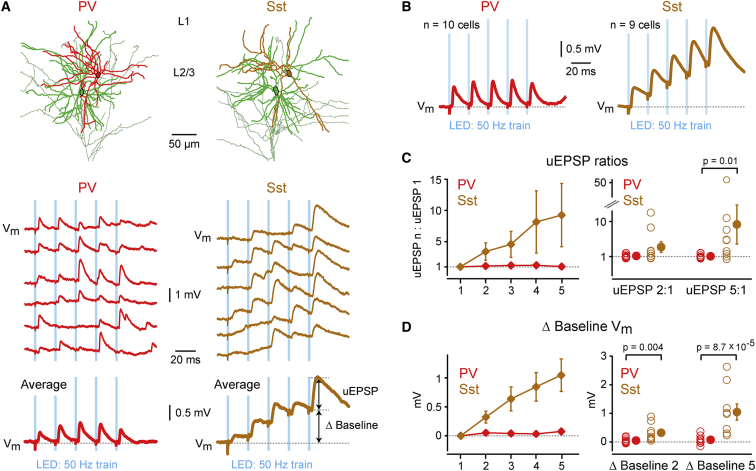
In Vivo Short-Term Synaptic Dynamics (A) Reconstruction of connected pairs of L2/3 Exc→PV and Exc→Sst neurons. Dendrites of the presynaptic excitatory neurons are colored in green, axons in gray. Dendrites of postsynaptic PV and Sst neurons are colored in red and brown, respectively. Example whole-cell recording of uEPSPs elicited in the PV (red) and Sst (brown) neuron during DOWN states by a 50 Hz train of five 1 ms light pulses. Single trial uEPSPs are shown above and average uEPSPs below. (B) Grand average uEPSPs for all connected PV and Sst neurons evoked by 50 Hz train of optogenetic stimuli during DOWN states. (C) Population uEPSP amplitude ratios comparing the amplitude of each uEPSP in the train to the amplitude of the first uEPSP for PV and Sst neurons (left). Individual neuron uEPSP amplitude ratios for uEPSP2 and uEPSP5 (right). Exc→Sst synapses facilitate, whereas Exc→PV synapses show little short-term dynamics. (D) Population difference in baseline V_m_ of each uEPSP in the train relative to the baseline V_m_ of the first uEPSP for PV and Sst neurons (left). Differences across individual neurons in baseline V_m_ at onset of uEPSP2 and uEPSP5 (right). uEPSPs summate prominently in Sst neurons, but not in PV neurons. Data are represented as mean ± SEM. Two-tail Wilcoxon rank-sum test assessed statistical significance. See also Figure S4.

The temporal pattern of presynaptic AP firing strongly influences excitatory synaptic transmission. We therefore measured in vivo uEPSP dynamics evoked by stimulating the presynaptic excitatory ChR2 neuron to fire a burst of five APs at 20 Hz (Figure S4) or 50 Hz (Figure 4A). At a stimulation frequency of 50 Hz, synapses targeting Sst neurons showed strong facilitation, whereas excitatory input to PV neurons showed a relatively reliable response with little short-term dynamics (uEPSP5 to uEPSP1 amplitude ratio: Sst 9.2 ± 5.0; PV 1.0 ± 0.2, mean ± SEM; p = 0.01) (Figures 4B and 4C). uEPSPs elicited in Sst neurons (but not PV neurons) also showed pronounced temporal summation, as measured by the depolarized baseline V_m_ at the onset of sequential uEPSPs (ΔBaseline V_m_ for uEPSP5: Sst 1.05 ± 0.28 mV; PV 0.08 ± 0.05 mV, mean ± SEM; p = 8.7 × 10^−5^) (Figures 4B and 4D).

## Discussion

By combining single-cell optogenetics with whole-cell V_m_ recordings, we systematically and directly quantified excitatory synaptic transmission onto PV- and Sst-expressing GABAergic neurons in L2/3 of the mouse barrel cortex in vivo. We found that PV and Sst neurons exhibit distinct intrinsic electrophysiological properties and receive local excitatory synaptic input with different connectivity, speed, reliability, and short-term dynamics in vivo. Our results extend current knowledge of cell-type-specific neuronal communication in vitro to the intact and spontaneously active neocortex in vivo.

### Single-Cell Optogenetics

Measurement of unitary postsynaptic potentials requires single APs to be precisely evoked in single presynaptic neurons. To date, this has been accomplished in electrophysiological recordings by injection of current either intracellularly or extracellularly during juxtacellular recording. Here, we show that single-cell electroporation of ChR2 provides an alternative method for precise stimulation with high reliability and low temporal jitter (Figure 2). Although high levels of ChR2 in axons could enhance calcium entry, thereby increasing neurotransmitter release probability in an unphysiological manner, our in vivo measurements of short-term plasticity rather suggest release probability lower than that expected from previous in vitro measurements using dual whole-cell recordings (see below). The optogenetic approach offers anatomical identification of the presynaptic neuron through expression of fluorescent proteins and allows long-term stimulation of the same neuron, tested here on the time scale of a few hours. The ability to stimulate the same neuron over long periods of time allows synaptic connectivity from the same presynaptic neuron to be assessed onto different potential postsynaptic neurons recorded sequentially (Figure 1). In future studies, it will be interesting to apply single-cell optogenetic stimulation paradigms to study behavioral effects of single-cell stimulation, which have so far been hampered by the short durations typically associated with intracellular and juxtacellular recordings in behaving animals (Houweling and Brecht, 2008).

### In Vivo versus In Vitro Measurements of Synaptic Connectivity

Cell-type-specific measurements of synaptic connectivity in the neocortex have so far been carried out in vitro in brain slice preparations. Axonal and dendritic arborisations are typically truncated during the preparation of brain slices, which could reduce the apparent measured synaptic connectivity. Here, we found that excitatory L2/3 pyramidal neurons in mouse barrel cortex in vivo provide synaptic input onto 51% (23/45) of nearby PV neurons (Figure 3). Closely related in vitro measurements from L2/3 barrel cortex found a similar connectivity of excitatory to PV neurons: mouse 58% (23/40) (Avermann et al., 2012) and rat 48% (19/40) (Kapfer et al., 2007). There is general agreement that synaptic connectivity is high from excitatory to PV cells (Holmgren et al., 2003; Hofer et al., 2011). The in vitro connectivity of excitatory and Sst neurons in rat L2/3 barrel cortex was determined to be 29% (Kapfer et al., 2007), in good agreement with our in vivo measurements of 31% (18/59) (Figure 3). However, there are also reports of higher levels of connectivity from excitatory to Sst L2/3 cells (Fanselow and Connors, 2010), and in rat L4 barrel cortex excitatory neurons were even found to connect preferentially to Sst compared to PV neurons (Beierlein et al., 2003). In addition to differences across cortical layers, it is also likely that synaptic connectivity will vary across cortical regions (Yoshimura and Callaway, 2005; Levy and Reyes, 2012).

### Properties of uEPSPs in PV and Sst Neurons Measured In Vivo

The uEPSPs measured in PV and Sst neurons had markedly different properties. On a trial-by-trial basis, the amplitude of uEPSPs had low variance and low failure rate in PV neurons, whereas the uEPSPs in Sst neurons had high variance and high failure rate. This suggests that the probability of releasing synaptic vesicles in response to an AP is lower for synapses onto Sst neurons (Buhl et al., 1997; Koester and Johnston, 2005). The clear distinction of failure and success trials in postsynaptic Sst neurons (Figures 1 and 3) presumably results from the very high input resistance of the Sst neurons (∼200 MΩ). The unreliable synaptic input to Sst neurons may contribute to the low correlation of V_m_ fluctuations in Sst neurons with the LFP, whereas PV neurons receive more reliable input from nearby excitatory neurons, thus giving high correlations with the LFP (Figure S3H). Differences in the properties of excitatory synaptic transmission might therefore contribute to the different V_m_ correlations of PV, Sst, and excitatory neurons in awake mice (Gentet et al., 2010, 2012).

The time course of the uEPSPs was also very different in PV and Sst neurons. The uEPSP rise time was faster in PV neurons compared to Sst neurons. The uEPSP duration was also much longer in Sst neurons compared to PV neurons. The different kinetics of the uEPSPs likely result from the intrinsic electrophysiological properties of the membrane time constants. PV neurons had a uEPSP decay time of 5.2 ms and a membrane time constant of 3.6 ms, whereas Sst neurons had a uEPSP decay time of 16.0 ms and a membrane time constant of 17.7 ms (Figure 3 and Tables S1 and S2). PV and Sst neurons have very little synaptic NMDA conductance (Matta et al., 2013), and excitation is therefore largely mediated by AMPA receptors, which typically evoke very brief synaptic conductances (∼2 ms). The membrane time constant therefore contributes importantly to the duration of the uEPSP measured at the soma.

PV neurons therefore appear to be designed for reliable and rapid signal processing, receiving brief, fast-rising uEPSPs with a low failure rate. In contrast, Sst neurons receive unreliable excitatory input and process it over much longer time scales, having long membrane time constants and therefore long-duration uEPSPs, which thus promote summation of uEPSPs (Figure 4D).

### Short-Term Synaptic Plasticity

We found that uEPSPs recorded in Sst neurons facilitated strongly in response to high-frequency stimulation of the presynaptic neuron (Figures 4 and S4). Our in vivo measurements are in good agreement with previous in vitro measurements showing strong short-term facilitation in postsynaptic Sst neurons (Reyes et al., 1998; Rozov et al., 2001; Beierlein et al., 2003; Koester and Johnston, 2005; Silberberg and Markram, 2007; Kapfer et al., 2007; Fanselow and Connors, 2010). The facilitation presumably results from the low release probability observed under baseline low-frequency stimulation, which allows for strong increases in release probability as calcium summates in the presynaptic boutons during high-frequency stimulation.

On the other hand, the reliable uEPSPs exhibiting little short-term plasticity in PV neurons that we found in vivo contrasts with the strongly depressing synaptic input typically reported for these neurons in vitro (Reyes et al., 1998; Rozov et al., 2001; Galarreta and Hestrin, 1998; Holmgren et al., 2003; Koester and Johnston, 2005; Kapfer et al., 2007; Hofer et al., 2011). Interestingly, direct comparison of synaptic transmission in vitro and in vivo at the calyx of Held also showed less synaptic depression in vivo due to elevated presynaptic firing rates in vivo, elevated neurotransmitter concentrations in vivo, and lower extracellular calcium concentrations in vivo compared to the typical values used in slice experiments (Lorteije et al., 2009).

### Synaptic Transmission across Cortical States—Future Perspectives

Although on average we did not find a consistent modulation of uEPSPs in PV or Sst neurons comparing quiescent cortical states (DOWN) and active cortical states (UP) (Figure 3H), in a few cells we found that uEPSP amplitude decreased significantly during UP states. Decreases in uEPSP amplitude during UP states (Crochet et al., 2005; Bruno and Sakmann, 2006) would be expected because the electrical driving force is different, with UP states being depolarized compared to DOWN states. In addition, the synaptic input occurring during UP states causes decreases in input resistance in some experimental preparations (Destexhe et al., 2003), but not others (Waters and Helmchen, 2006; Mateo et al., 2011). On the other hand, depolarization can also enhance presynaptic neurotransmitter release (Shu et al., 2006) and activate postsynaptic voltage-gated somatic and dendritic conductances, which could boost uEPSP amplitude. The regulation of synaptic transmission across cortical states may therefore be complicated and deserves further detailed investigation. It is also possible that anesthesia directly affects synaptic transmission. In future experiments, it will therefore be important to extend these first in vivo measurements of cell-type-specific synaptic transmission to other well-defined neocortical cell types and to compare synaptic transmission across different behavioral states in awake mice.

## Experimental Procedures

All experiments were carried out in accordance with protocols approved by the Swiss Federal Veterinary Office (see Supplemental Experimental Procedures).

## Supplemental Information

Document S1. Supplemental Experimental Procedures, Figures S1–S4, and Tables S1 and S2

Movie S1. Single-Cell In Vivo Electroporation of DNA Encoding eGFP and a Fast Variant of ChR2 Together with Alexa 488 Imaged Using a Two-Photon Microscope, Related to Figure 1 Positive pressure inside the electrode ejects green fluorescent dye (Alexa 488) and helps maintain the tip of the electrode clean. Unlabeled neurons in L2/3 are visualized as shadows. Upon electrode contact with the cell soma, a train of −12 V pulses each lasting 0.5 ms at a frequency of 50 Hz for 1 s delivers the DNA encoding eGFP and ChR2 together with Alexa 488 to the targeted neuron. Red fluorescence is from tdTomato-expressing neurons in the Sst-Cre × LSL-tdTomato mouse.

Document S2. Article plus Supplemental Information
