# Supplementary material for: In Vivo Measurement of Cell-Type-Specific Synaptic Connectivity and Synaptic Transmission in Layer 2/3 Mouse Barrel Cortex
Source: Neuron. 2015 Jan 7;85(1):68–75. doi: 10.1016/j.neuron.2014.11.025 (PMC4305188; doi:10.1016/j.neuron.2014.11.025)
Supplement: Document S1. Supplemental Experimental Procedures, Figures S1–S4, and Tables S1 and S2 [file mmc1.pdf]

Neuron, Volume *85*

Supplemental Information

In Vivo Measurement of Cell-Type-Specific Synaptic Connectivity and Synaptic Transmission in Layer 2/3 Mouse Barrel Cortex

Aurélie Pala and Carl C.H. Petersen

Figure S1

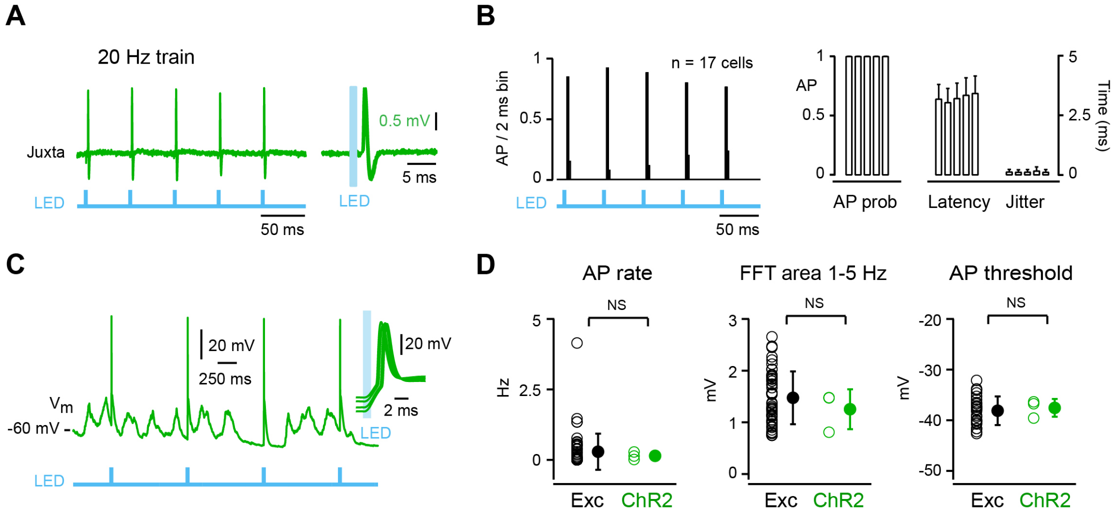

Figure S1. Optogenetic control of action potential firing of a single excitatory neuron in vivo, related to Figure 2.

(A) Example single APs elicited by an optogenetic stimulus made of a 20 Hz train of five 1-ms light pulses recorded juxtacellularly in the same L2/3 ChR2-expressing excitatory neuron as in Figure 2.

(B) Population peristimulus time histogram of light-evoked AP timing *(left)* and light-evoked AP probability, latency and jitter *(right)* for an optogenetic stimulus made of a 20 Hz train of five 1-ms light pulses.

(C) Example whole-cell recording of single APs elicited by single 1-ms light pulses delivered at 1 Hz in a different ChR2-expressing excitatory neuron than in (A). Inset shows magnified APs shape.

(D) Spontaneous AP rate, V_m 1-5 Hz FFT amplitude and AP threshold were similar in ChR2-expressing excitatory neurons (n = 3 cells) compared to non-expressing excitatory neurons.

Data are represented as mean ± SD. Two-tail Wilcoxon rank-sum test assessed statistical significance.

Figure S2

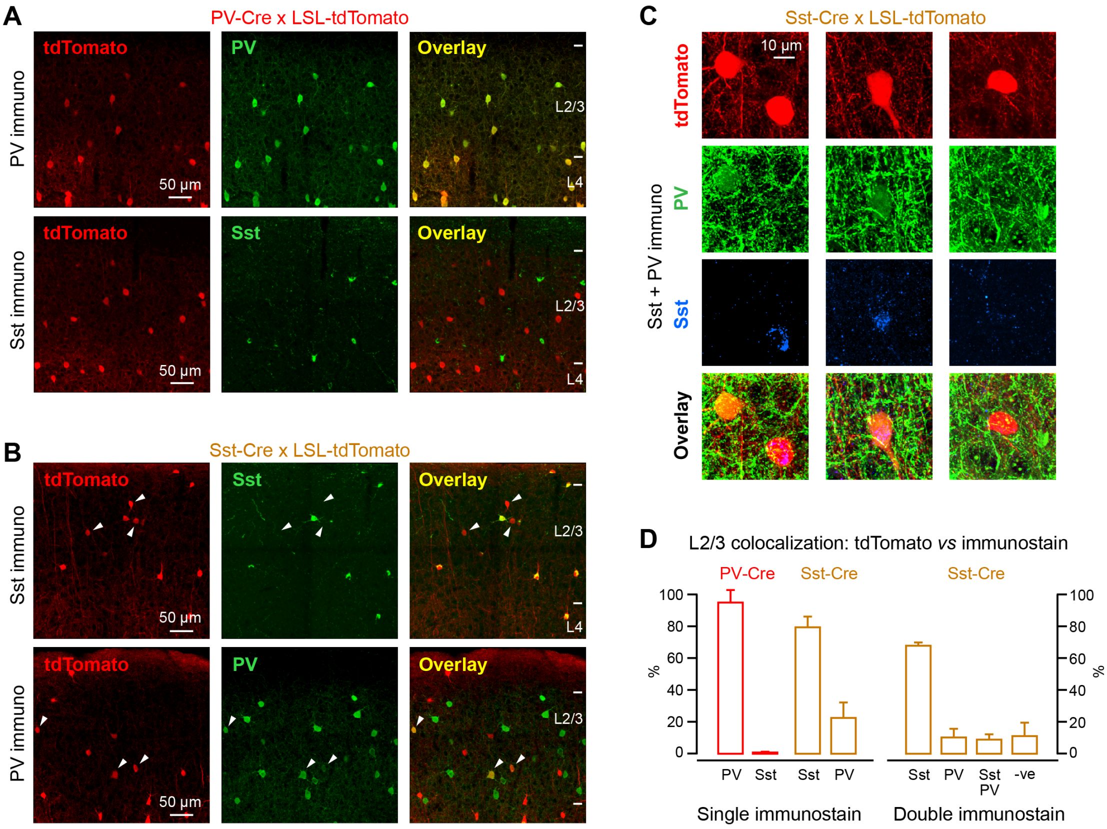

Figure S2. Immunostaining against PV and Sst in barrel cortex of PV-Cre x LSL-tdTomato and Sst-Cre x LSL-tdTomato mice, related to Figure 3.

(A) Example of single immunostaining against PV or Sst (green) in barrel cortex upper layers of a PV-Cre x LSL-tdTomato mouse (red).

(B) Same as in (A) but for a Sst-Cre x LSL-tdTomato mouse. Some L2/3 tdTomato-expressing neurons are not positive for Sst (*above*, arrows) while some L2/3 tdTomato-expressing neurons are positive for PV (*below*, arrows).

(C) Example of double immunostaining against both PV (green) and Sst (blue) in a Sst-Cre x LSL-tdTomato mouse (red). Some L2/3 tdTomato-expressing neurons are positive only for Sst while some are positive only for PV (*left*), some are positive for both Sst and PV (*middle*) and some are negative for both PV and Sst (*right*).

(D) In L2/3 barrel cortex of PV-Cre x LSL-tdTomato mice 95.6 ± 7.6 % (n = 270 cells across 3 mice) of tdTomato-expressing neurons express PV while 0.8 ± 0.7 % (n = 284 cells across 3 mice) express Sst (red, *left*). In L2/3 barrel cortex of Sst-Cre x LSL-tdTomato mouse 80.0 ± 6.5 % (n = 157 cells across 3 mice) of tdTomato-expressing neurons express Sst while 23.0 ± 9.5 % (n = 181 cells across 3 mice) express PV (brown, *left*). Double immunostaining (*right*) shows that 68.4 ± 1.8 % of Sst-tdTomato neurons express Sst only, 10.7 ± 5.3 % express PV only, 9.4 ± 3.1 %

express both Sst and PV, and 11.5 ± 8.2 % express neither PV nor Sst (n = 181 cells across 3 mice).

Data are represented as mean ± SD.

Figure S3

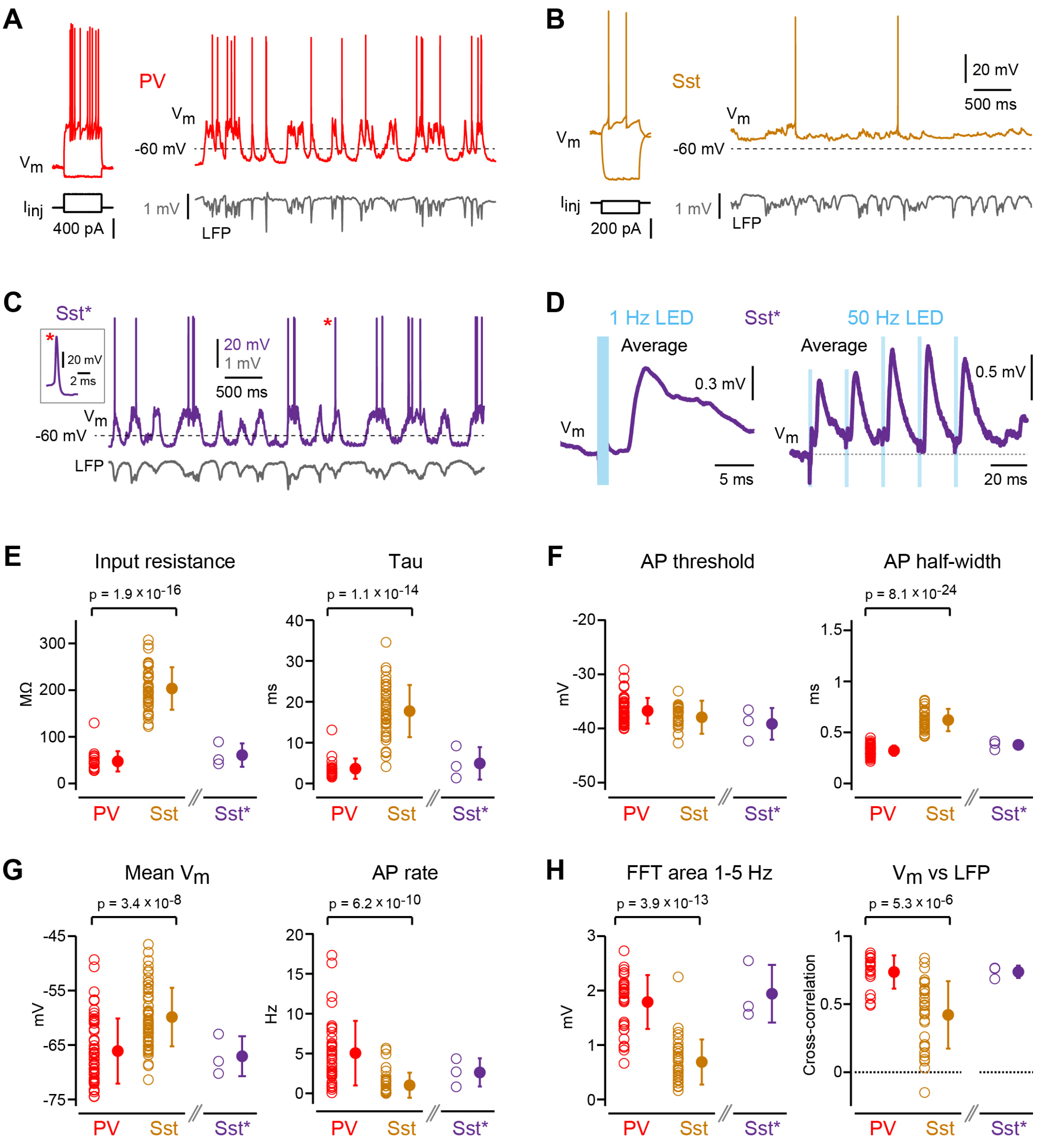

Figure S3. Distinct electrophysiological properties of PV and Sst neurons in L2/3 mouse barrel cortex in vivo, related to Figure 3.

(A) Example whole-cell recording of rheobase AP firing and spontaneous V_m dynamics together with LFP recording for a PV neuron.

(B) Same as in (A) but for a Sst neuron.

(C) A small subset of Sst neurons (Sst*, n = 3 out of 66 recorded Sst-Cre x tdTomato neurons) with distinct electrophysiological properties are excluded from the Sst dataset because they are likely to be PV neurons. Example whole-cell recording of a Sst* neuron. Inset shows magnified AP shape. This Sst* neuron shows large V_m slow-wave oscillation amplitude and relatively high firing rate with a narrow and fast AP waveform, similar to PV neurons.

(D) Average uEPSPs elicited during DOWN state in the same Sst* neuron as in (C) by a 1-ms light pulse *(left)* and by a 50 Hz train of five 1-ms light pulses *(right)*. This Sst* neuron displays uEPSP with fast kinetics, little short-term dynamics and little summation, reminiscent of PV neurons.

(E) Input resistance and membrane time constant (Tau) are smaller in PV neurons compared to Sst neurons.

(F) AP threshold is similar for PV and Sst neurons. AP half-width is shorter in PV neurons compared to Sst neurons.

(G) Mean V_m is more hyperpolarized in PV neurons compared to Sst neurons. Spontaneous AP rate is higher in PV neurons compared to Sst neurons.

(H) The FFT of the V_m integrated over 1-5 Hz and cross-correlation between V_m and LFP at zero-time-lag are both larger in PV neurons compared to Sst neurons.

Data are represented as mean ± SD. Two-tail Wilcoxon rank-sum test assessed statistical significance. See also Table S1.

Figure S4

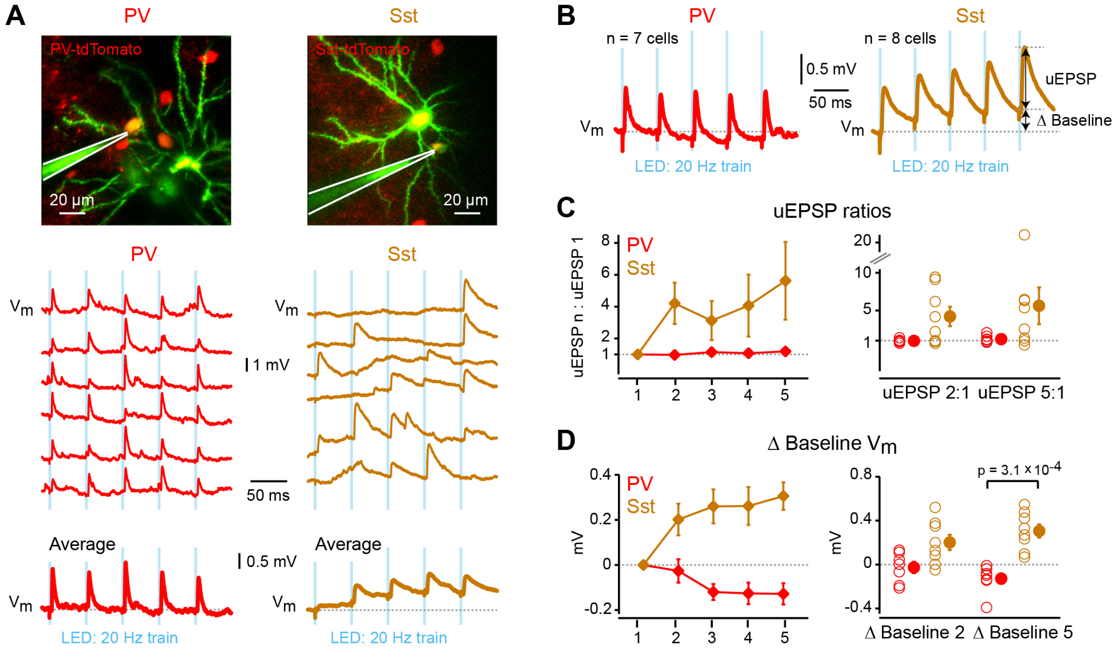

Figure S4. In vivo short-term synaptic dynamics for 20 Hz train of optogenetic stimuli, related to Figure 4.

(A) Example whole-cell recording of uEPSPs elicited in a PV (red) and Sst (brown) neuron during DOWN states by an optogenetic stimulus made of a 20 Hz train of five 1-ms light pulses. The neurons represented here are different than those in Figure 4. Single trial uEPSPs are shown above and average uEPSPs below. The in vivo two-photon images show the whole-cell recording pipette (Alexa 488 dye, green), the recorded tdTomato-expressing neuron (yellow) and part of the presynaptic eGFP- and ChR2-expressing neuron (green).

(B) Grand average uEPSPs for all connected PV and Sst neurons evoked by 20 Hz train of optogenetic stimuli during DOWN states.

(C) Population uEPSP amplitude ratios comparing the amplitude of each uEPSP in the train to the amplitude of the first uEPSP for PV and Sst neurons *(left)*. Individual neurons uEPSP amplitude ratios for uEPSP2 and uEPSP5 (*right*).

(D) Population difference in baseline V_m of each uEPSP in the train relative to the baseline V_m of the first uEPSP for PV and Sst neurons *(left)*. Differences across individual neurons in baseline V_m at onset of uEPSP2 and uEPSP5 *(right)*. uEPSPs summate in Sst neurons, but not in PV neurons.

Data are represented as mean ± SEM. Two-tail Wilcoxon rank-sum test assessed statistical significance.

Table S1

Properties	PV	Sst	Exc
Input resistance (MΩ)	**47 ± 22 (n = 21)**	**203 ± 45 (n = 43)**	**47 ± 13 (n = 27)**
Tau (ms)	**3.6 ± 2.5 (n = 21)**	**17.7 ± 6.4 (n = 45)**	**6.5 ± 1.6 (n = 27)**
Mean V_m (mV)	**-66.1 ± 6.0 (n = 52)**	**-59.9 ± 5.4 (n = 63)**	**-68.7 ± 5.6 (n = 54)**
AP rate (Hz)	**5.05 ± 4.05 (n = 38)**	**1.02 ± 1.56 (n = 37)**	**0.29 ± 0.64 (n = 54)**
AP threshold (mV)	**-36.7 ± 2.4 (n = 51)**	**-37.9 ± 3.0 (n = 33)**	**-38.1 ± 2.8 (n = 25)**
AP half-width (ms)	**0.32 ± 0.05 (n = 51)**	**0.62 ± 0.11 (n = 33)**	**1.05 ± 0.16 (n = 25)**
FFT area 1-5 Hz (mV)	**1.79 ± 0.50 (n = 35)**	**0.69 ± 0.41 (n = 35)**	**1.47 ± 0.51 (n = 49)**
V_m vs LFP cross-correlation	**0.74 ± 0.12 (n = 19)**	**0.42 ± 0.25 (n = 33)**	**0.71 ± 0.10 (n = 45)**

Table S1. Electrophysiological properties of parvalbumin-expressing GABAergic neurons (PV), somatostatin-expressing GABAergic neurons (Sst) and excitatory neurons (Exc), related to Figure 3.

Table S2

uEPSP property	PV	Sst	uEPSP property	PV	Sst
**Amplitude (mV)**	**n = 25**	**n = 17**	**Rise time (ms)**	**n = 25**	**n = 17**
mean ± SD	0.53 ± 0.39	0.50 ± 0.86	mean ± SD	0.68 ± 0.32	1.76 ± 1.40
median	0.39	0.21	median	0.61	1.31
range	0.03 – 1.40	0.02 – 3.48	range	0.33 – 1.85	0.58 – 5.89
**Failure rate (%)**	**n = 22**	**n = 16**	**Half-width (ms)**	**n = 24**	**n = 13**
mean ± SD	27.4 ± 15.5	68.0 ± 29.1	mean ± SD	4.0 ± 1.4	11.6 ± 6.7
median	31.7	80.2	median	4.3	9.1
range	0 – 51.0	4.5 – 90.3	range	1.2 – 6.6	3.9 – 23.1
**Coefficient of variation**	**n = 23**	**n = 16**	**Tau decay (ms)**	**n = 21**	**n = 9**
mean ± SD	0.33 ± 0.28	0.92 ± 0.53	mean ± SD	5.2 ± 3.0	16.0 ± 8.5
median	0.32	0.88	median	4.8	15.2
range	-0.31 – 0.94	0.26 – 1.92	range	1.3 – 12.7	6.0 – 32.9

Table S2. Properties of unitary EPSPs (uEPSP) recorded in parvalbumin-expressing GABAergic neurons (PV) and somatostatin-expressing GABAergic neurons (Sst), related to Figure 3.

Movie S1

Movie S1. Single-cell in vivo electroporation of DNA encoding eGFP and a fast variant of ChR2 together with Alexa 488 imaged using a two-photon microscope, related to Figure 1.

Positive pressure inside the electrode ejects green fluorescent dye (Alexa 488) and helps maintain the tip of the electrode clean. Unlabeled neurons in L2/3 are visualized as shadows. Upon electrode contact with the cell soma, a train of -12 V pulses each lasting 0.5 ms at a frequency of 50 Hz for 1 s delivers the DNA encoding eGFP and ChR2 together with Alexa 488 to the targeted neuron. Red fluorescence is from tdTomato-expressing neurons in the Sst-Cre x LSL-tdTomato mouse.

Supplemental Experimental Procedures

Animal preparation and surgery

All experiments were carried out with 4-8 week old female and male PV-IRES-Cre (Hippenmeyer et al., 2005) or Sst-IRES-Cre (Taniguchi et al. 2011) mice crossed with CAG-Lox-STOP-Lox-tdTomato (LSL-tdTomato) reporter mice (Madisen et al., 2010) in accordance with protocols approved by the Swiss Federal Veterinary Office. Mice were maintained under 1-2% isoflurane anesthesia while being implanted with a custom-made head-holder and a recording chamber. The location of the left C2 barrel column was functionally identified through intrinsic optical imaging under 0.5 - 1% isoflurane anesthesia (Lefort et al., 2009) and a small craniotomy (diameter 0.5 - 1 mm) was made taking care to leave the dura intact.

Single-cell electroporation

Electroporation of a single non-tdTomato neuron per PV-Cre x LSL-tdTomato or Sst-Cre x LSL-tdTomato mouse was carried out under 1% isoflurane anesthesia with slight modifications from a previously described protocol (Kitamura et al., 2008). In brief, a glass pipette with a resistance of 10-17 MΩ was filled with the same solution used for whole-cell recordings (see below) to which Alexa 488 dye (50-100 μM) (Invitrogen), pCAG-eGFP plasmid DNA (100 ng/μl) (Addgene plasmid 11150, kindly provided by Connie Cepko) (Matsuda and Cepko, 2004) and pCI-hSynapsin-ChR2(E123T/T159C) (200 ng/μl) (kindly provided by Thomas Oertner) (Berndt et al., 2011) were added. A two-photon microscope (Prairie Technologies) was used to visualize the pipette and the tdTomato-negative cell somas as dark shadows over a brighter background. The pipette was inserted in the brain through the intact dura and brought into close contact with the cell body of the target neuron and 50 pulses of negative voltage step (0.5 ms, –12 V) were delivered at 50 Hz using a pulse generator (Axoporator 800A, Molecular Devices). The craniotomy was then covered with a silicone elastomer (Kwik-Cast, WPI) and the mice were returned to their home cage for 24 hours before proceeding to electrophysiological recordings.

Electrophysiology

24 hours after electroporation, mice were re-anesthetized with 1-2% isoflurane and the dura was partially removed. Mice were placed again under the two-photon microscope and kept under 0.8-1.5% isoflurane anesthesia. The location of the single ChR2-expressing neuron was identified by the cortical blood vasculature pattern and its excitatory nature was confirmed by its overall morphology and the presence of numerous dendritic spines. Local field potential (LFP) was continuously recorded with a 2-4 MΩ glass pipette filled with Ringer solution containing 10-25 µM Alexa 594 dye and lowered at a depth of 150-250 µm from the pia and within 250 µm from the ChR2-expressing neuron. Two-photon targeted juxtacellular recording of the ChR2-expressing neuron was performed with 4-6 MΩ glass pipettes filled with the same solution as used for LFP recordings. Two-photon targeted whole-cell patch-clamp recordings were performed as previously described (Margrie et al. 2003, Gentet et al. 2010, Mateo et al. 2011). 5-7 MΩ glass pipettes were filled with a solution containing (in mM): 135 potassium gluconate, 4 KCl, 10 HEPES, 10 sodium phosphocreatine, 4 MgATP, 0.3 Na_3GTP (adjusted to pH 7.3 with KOH), to which 25-75 µM Alexa 488 dye and 3 mg/ml biocytin were added. Patch-clamp recordings were obtained in current-clamp mode and V_m was not corrected for liquid junction potentials. When current was injected to characterize intrinsic electrophysiological properties, series resistance subtraction was performed offline (see below). All recorded signals were amplified by a Multiclamp 700B amplifier (Axon Instruments), Bessel filtered at 10 kHz and digitized at 20 kHz by an ITC-18 (Instrutech Corporation) under the control of a custom program written in IgorPro (Wavemetrics).

Optogenetic stimulation

A collimated 470 nm superbright LED (Luxeon, Philips) was placed at the back of the two-photon objective to generate wide field stimulation. Optogenetic stimulus consisted of either a single square pulse of light of 1-ms duration and 10-70 mW/mm^2 intensity (mean ± SD: 32 ± 23 mW/mm^2), delivered with an interval of 1 s, or of a 20 or 50 Hz train of five 1-ms light pulses of similar intensity, delivered with a minimum interval of 5 s. A constant 470 nm background illumination made of an array of small LEDs (Everlight Electronics) was placed in front of the mouse during some recording sessions.

Histology and immunohistochemistry

After termination of the recordings, some mice were perfused with a 4% paraformaldehyde (PFA) solution, made by diluting a 32% PFA solution (EMS) in 0.1 M PBS. Brains were post-fixed for maximum 2 hours in the same solution, which was then replaced by a 0.1 M PBS solution. 50 µm thick coronal sections were cut using a semi-automated vibratome (VT1000S, Leica). Primary antibody against eGFP (rabbit, 1:5000, Abcam, Ab290) followed by secondary anti-rabbit antibody coupled to Alexa 488 (donkey, 1:200, Invitrogen) were used to enhance the eGFP signal of presynaptic neurons. Streptavidin coupled to Alexa 647 (1:2000, Invitrogen) was used to reveal biocytin filling of postsynaptic neurons. Images were obtained with a laser scanning confocal microscope (LSM 700, Zeiss) equipped with an oil-immersion 63x/1.4NA objective. Three-dimensional anatomical reconstructions were traced from confocal fluorescence image stacks using Neurolucida (MBF Bioscience).

Three 8-week old PV-Cre x LSL-tdTomato mice and three 8-week old Sst-Cre x LSL-tdTomato mice were used for single immunostains against parvalbumin (PV) or somatostatin (Sst), which were performed after a similar perfusion, post-fixation and slicing procedure as described above. Primary antibody against PV (rabbit, 1:1000, Swant, PV28) or primary antibody against Sst (rat, 1:200, Millipore, Mab354) were used. Secondary antibodies were goat anti-rabbit or goat anti-rat coupled to Alexa 647 (1:500, Invitrogen).

Three 7-week old Sst-Cre x LSL-tdTomato mice were used for double immunohistochemistry against both PV and Sst. Both primary and secondary antibodies to reveal Sst were the same as the one used for single immunohistochemistry. Primary antibody to reveal PV was also similar while secondary goat anti-rabbit coupled to Alexa 405 was used (1:200, Invitrogen). Images were obtained with the same laser scanning confocal microscope as above and either a 40x/1.3NA or a 63x/1.4NA oil-immersion objective. 405 nm, 555 nm and 639 nm solid state lasers were used to excite Alexa 405, tdTomato and Alexa 647 respectively. A 450 nm dichroic mirror followed by a 490 nm short pass filter were used to collect Alexa 405 fluorescence. A 500 nm dichroic mirror followed by a 505-600 nm band pass filter were used to collect tdTomato fluorescence. Alexa 647 fluorescence was collected using a 630 nm dichroic mirror followed by a 640 nm long pass filter. Colocalization of the markers with tdTomato fluorescence was assessed by careful inspection of the image stacks.

Data analysis

To assess state-specific optogenetic control of AP firing in ChR2-expressing excitatory neurons, periods of DOWN and UP state were identified through the simultaneously recorded LFP. In brief, the LFP was band pass filtered between 0.1 and 200 Hz and a sliding FFT (window size: 150 ms, overlap: 125 ms) was computed. Principal component analysis of the real part of the FFT followed by a Gaussian mixture model were used to extract and classify the twenty-five most relevant LFP frequency features of each window into three clusters, corresponding to UP, DOWN and transition states. An optogenetic stimulus was considered as occurring during DOWN state if the window before it and the second one after it were classified as belonging to the DOWN cluster. Similarly, a stimulus was considered as occurring during UP state if the window before it and the second one after it were classified as belonging to the UP cluster. APs were considered as optogenetically triggered if their peak happened within 20 ms of the end of the 1 ms light stimulus. AP latency was defined as the time elapsed between light stimulus onset and AP peak time. AP jitter was defined as the standard deviation of the AP latency. Each metric was separately computed for DOWN and UP states.

To investigate connectivity between pairs of neurons, initial analysis focused on optogenetic stimuli occurring during DOWN state only. DOWN and UP states were mostly identified using a double-threshold method applied directly on the V_m of individually recorded PV and Sst neurons. For PV neurons, the two thresholds were defined as the most hyperpolarized V_m value of the given recording sweep plus 5 mV, and plus 10 mV for threshold 1 and threshold 2 respectively. For Sst neurons, 3.5 mV and 5.5 mV were added to the most hyperpolarized V_m value of the given recording sweep to define threshold 1 and threshold 2. An optogenetic stimulus was considered as occurring during DOWN state if both the mean V_m averaged during the 10 ms preceding light onset and the mean V_m averaged during a time window ranging from 30 to 40 ms after light onset were smaller than threshold 1. Similarly, an optogenetic stimulus was considered as occurring during UP state if both average V_m values were larger than threshold 2. A subset of Sst neurons displayed minimal spontaneous V_m fluctuations, precluding the use of the double-threshold method. In such cases, DOWN and UP state identification was performed using the simultaneously recorded LFP as described above. A stimulus-triggered V_m average was computed for all optogenetic stimuli occurring during DOWN states (mean ± SD: 83 ± 43 stimuli; median: 82 stimuli) and compared with averaged spontaneous DOWN state V_m fluctuations. Recordings with less than 20 light stimuli occurring

during DOWN states were not considered for connectivity analysis. Neurons were considered to be synaptically connected if there was a clear difference between the average V_m traces with and without optogenetic stimulation, and that the timecourse was consistent with that of postsynaptic potentials. Specifically, we compared average V_m traces with and without optogenetic stimulation within a peak search-window, whose location and size was defined by the presynaptic ChR2-expressing neuron AP firing properties. It was set as the timing from "AP latency – AP jitter + 1 ms" to "AP latency + AP jitter + 3 ms" after light onset for PV neurons (peak-search window size: 3.1 ± 1.5 ms, mean ± SD) and that from "AP latency – AP jitter + 1 ms" to "AP latency + AP jitter + 5 ms" for Sst neurons (peak-search window size: 4.8 ± 0.5 ms, mean ± SD). Stimulus-triggered V_m averages of 2 PV neurons and 1 Sst neuron computed from less than 20 optogenetic stimuli were nonetheless considered for further uEPSP properties analysis, as they displayed clear synaptic connections.

Stimulus-triggered V_m average was used to quantify uEPSP amplitude and rise-time. uEPSP amplitude was calculated as the difference between the mean V_m averaged over a 0.5 ms window centered at the peak of the uEPSP and the mean baseline V_m averaged over a 1 ms window taken after the end of the optogenetic stimulus. uEPSP rise time corresponded to the time elapsed from 20% to 80% of the amplitude on the rising phase of the averaged uEPSP. uEPSP half-width and decay time constant (Tau decay) were extracted from a light stimulus-triggered V_m average made of a subset of trials elicited during DOWN states, which contained no major spontaneous V_m fluctuations from 10 to 20 ms after light stimulus onset. uEPSP half-width was calculated as the full width duration of the uEPSP at half of its maximum amplitude. Tau decay was determined by fitting a single exponential on the decaying phase of the averaged uEPSP, starting 1 ms after the peak and 2 ms after the peak for PV neurons and Sst neurons respectively. Synaptic transmission failure rate was estimated by calculating the fraction of all trials occurring during DOWN states where a clear uEPSP could not be detected within the same peak-search window as used to assess the presence or absence of a synaptic connection. To compute uEPSP amplitude coefficient of variation including failures, single trial uEPSP amplitude was measured as the difference between the mean V_m averaged over a 0.5 ms window centered at the peak of the uEPSP detected within the peak-search window (see above) and the mean baseline V_m averaged over a 1 ms window taken after the end of the optogenetic stimulus. Standard deviation of a similarly computed amplitude distribution for four DOWN state time points at which no light stimuli were applied was subtracted from the standard deviation of the obtained uEPSP amplitude distribution before dividing it by its mean in order to correct for spontaneous V_m

fluctuations occurring during DOWN states (Feldmeyer et al. 1999; Lefort et al. 2009).

For UP – DOWN state comparison of uEPSP amplitude, a light stimulus-triggered average of the V_m was obtained for UP and DOWN states separately. UP state trials where postsynaptic APs were present during a 30 ms (PV neurons) or a 50 ms (Sst neurons) time window starting 10 ms before light stimulus onset were omitted from the stimulus-triggered V_m average. Recordings with less than 20 optogenetic stimuli in either UP or DOWN states were not included in the analysis. UP state uEPSP amplitude was computed as described for DOWN state uEPSP amplitude. Peak-search window size was adjusted to match presynaptic ChR2-expressing neuron light-evoked AP firing properties during UP states.

To measure short-term synaptic dynamics, a stimulus-triggered average of the V_m was computed from all 20 Hz or 50 Hz optogenetic stimuli where the five light stimuli of the train occurred during DOWN states. Amplitudes of averaged uEPSPs during the stimulus train were analyzed as for single light pulses, except for the second to fifth uEPSPs in Sst neurons, where baseline V_m was extracted from the value taken by a single exponential fit of the decaying phase of the preceding uEPSP (fit start 2 ms after uEPSP peak) at the time of the current uEPSP peak.

Euclidean distance between the soma of the ChR2-expressing excitatory presynaptic neuron and the soma of the PV or Sst postsynaptic neuron was computed from two-photon image stacks acquired in vivo.

Electrophysiological properties of PV and Sst neurons were quantified as follows. Input resistance and membrane time constant (Tau) were measured by repeated current injections (-100 pA, 500 ms) immediately after establishing whole-cell configuration. Average V_m response was fitted offline with a double exponential from 0.4 ms to 50 ms after the onset of the current injection to determine and subtract the early fast component of the response, due to series resistance. Input resistance was calculated as the difference in the corrected mean V_m averaged over two 100 ms periods (one immediately before current injection and the other at the end of current injection) divided by the amount of injected current. Tau was determined by fitting the V_m with a single exponential from 1 ms to 60 ms after the onset of current injection. AP threshold was defined as the V_m at which the slope of rise of the voltage crossed 50 V/s (Kole and Stuart 2008). AP half-width was computed as the full width of the AP at half of its maximum amplitude measured from threshold to peak. Mean V_m and V_m FFT amplitude were computed across 20 s

sweeps of recording encompassing both UP and DOWN states. Spontaneous AP rate was computed across UP and DOWN states for the whole duration of the recording. To compute V_m *vs* LFP cross-correlation, V_m was offset by its average value and normalized by its standard deviation, and LFP was band pass filtered between 0.3 and 200 Hz.

Population data are represented as mean ± SD (except for Figures 4 and S4, where mean ± SEM is shown). Two-tail Wilcoxon rank-sum and signed-rank tests were used to compare two groups of unpaired and paired data respectively. χ^2 test was used to assess significant differences in connectivity rate. Spearman's ρ was used to quantify monotonic correlation between uEPSP amplitude and failure rate. Pearson's r was used to test for a linear relationship between connectivity rate and intersomatic distance. Data analysis was carried out in IgorPro (Wavemetrics) and Matlab (Mathworks) and statistical analysis was performed in Matlab.

Supplemental References

Feldmeyer, D., Egger, V., Lubke, J., and Sakmann, B. (1999). Reliable synaptic connections between pairs of excitatory layer 4 neurones within a single 'barrel' of developing rat somatosensory cortex. J. Physiol. *521*, 169-190.

Hippenmeyer, S., Vrieseling, E., Sigrist, M., Portmann, T., Laengle, C., Ladle, D.R., and Arber, S. (2005). A developmental switch in the response of DRG neurons to ETS transcription factor signaling. PLoS Biol. *3*, e159.

Kole, M.H., and Stuart, G.J. (2008). Is action potential threshold lowest in the axon? Nat. Neurosci. *11*, 1253-1255.

Madisen, L., Zwingman, T.A., Sunkin, S.M., Oh, S.W., Zariwala, H.A., Gu, H., Ng, L.L., Palmiter, R.D., Hawrylycz, M.J., Jones, A.R., et al. (2010). A robust and high-throughput Cre reporting and characterization system for the whole mouse brain. Nat. Neurosci. *13*, 133-140.

Margrie, T.W., Meyer, A.H., Caputi, A., Monyer, H., Hasan, M.T., Schaefer, A.T., Denk, W., and Brecht, M. (2003). Targeted whole-cell recordings in the mammalian brain in vivo. Neuron *39*, 911-918.

Matsuda, T., and Cepko, C.L. (2004). Electroporation and RNA interference in the rodent retina in vivo and in vitro. Proc. Natl. Acad. Sci. USA *101*, 16-22.

Taniguchi, H., He, M., Wu, P., Kim, S., Paik, R., Sugino, K., Kvitsiani, D., Fu, Y., Lu, J., Lin, Y., et al. (2011). A resource of Cre driver lines for genetic targeting of GABAergic neurons in cerebral cortex. Neuron *71*, 995-1013.
