# Supplementary figures and images for: In Vivo Measurement of Cell-Type-Specific Synaptic Connectivity and Synaptic Transmission in Layer 2/3 Mouse Barrel Cortex
Source: Neuron. 2015 Jan 7;85(1):68–75. doi: 10.1016/j.neuron.2014.11.025 (PMC4305188; doi:10.1016/j.neuron.2014.11.025)

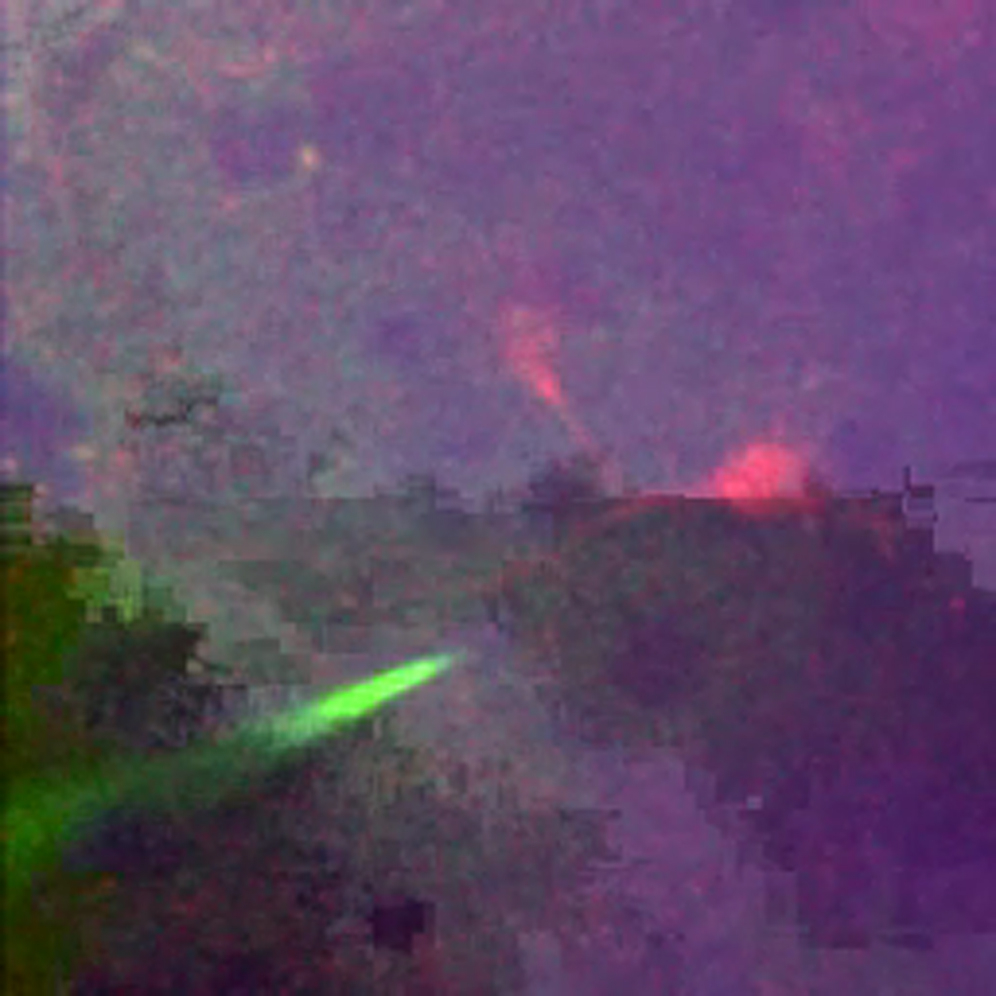

Supplement: Movie S1. Single-Cell In Vivo Electroporation of DNA Encoding eGFP and a Fast Variant of ChR2 Together with Alexa 488 Imaged Using a Two-Photon Microscope, Related to Figure 1 — Positive pressure inside the electrode ejects green fluorescent dye (Alexa 488) and helps maintain the tip of the electrode clean. Unlabeled neurons in L2/3 are visualized as shadows. Upon electrode contact with the cell soma, a train of −12 V pulses each lasting 0.5 ms at a frequency of 50 Hz for 1 s delivers the DNA encoding eGFP and ChR2 together with Alexa 488 to the targeted neuron. Red fluorescence is from tdTomato-expressing neurons in the Sst-Cre × LSL-tdTomato mouse. [file mmc2.jpg]
