# Supplementary material for: In Vivo Measurement of Cell-Type-Specific Synaptic Connectivity and Synaptic Transmission in Layer 2/3 Mouse Barrel Cortex
Source: Neuron. 2015 Jan 7;85(1):68–75. doi: 10.1016/j.neuron.2014.11.025 (PMC4305188; doi:10.1016/j.neuron.2014.11.025)
Supplement: Document S2. Article plus Supplemental Information [file mmc3.pdf]

Neuron

Report

In Vivo Measurement of Cell-Type-Specific Synaptic Connectivity and Synaptic Transmission in Layer 2/3 Mouse Barrel Cortex

Highlights

- Single-cell optogenetics for precise stimulation of action potentials in vivo
- In vivo whole-cell recordings from genetically defined postsynaptic GABAergic neurons
- Parvalbumin-expressing neurons receive strong, fast, and reliable excitatory input
- Somatostatin-expressing neurons receive longer-lasting, facilitating excitatory input

Authors

Aurélie Pala, Carl C.H. Petersen

Correspondence

carl.petersen@epfl.ch

In Brief

Pala and Petersen use single-cell optogenetics and two-photon targeted whole-cell recordings to measure synaptic connectivity and synaptic transmission in vivo from excitatory neurons onto parvalbumin-expressing and somatostatin-expressing GABAergic neurons in layer 2/3 of mouse barrel cortex.

Pala & Petersen, 2015, Neuron *85*, 68–75
January 7, 2015 ©2015 The Authors
http://dx.doi.org/10.1016/j.neuron.2014.11.025

Neuron
Report

In Vivo Measurement of Cell-Type-Specific Synaptic Connectivity and Synaptic Transmission in Layer 2/3 Mouse Barrel Cortex

Aurélie Pala[1] and Carl C.H. Petersen[1,*]
[1]Laboratory of Sensory Processing, Brain Mind Institute, Faculty of Life Sciences, École Polytechnique Fédérale de Lausanne (EPFL), Lausanne 1015, Switzerland
*Correspondence: carl.petersen@epfl.ch
http://dx.doi.org/10.1016/j.neuron.2014.11.025
This is an open access article under the CC BY license (http://creativecommons.org/licenses/by/3.0/).

SUMMARY

Intracellular recordings of membrane potential in vitro have defined fundamental properties of synaptic communication. Much less is known about the properties of synaptic connectivity and synaptic transmission in vivo. Here, we combined single-cell optogenetics with whole-cell recordings to investigate glutamatergic synaptic transmission in vivo from single identified excitatory neurons onto two genetically defined subtypes of inhibitory GABAergic neurons in layer 2/3 mouse barrel cortex. We found that parvalbumin-expressing (PV) GABAergic neurons received unitary glutamatergic synaptic input with higher probability than somatostatin-expressing (Sst) GABAergic neurons. Unitary excitatory postsynaptic potentials onto PV neurons were also faster and more reliable than inputs onto Sst neurons. Excitatory synapses targeting Sst neurons displayed strong short-term facilitation, while those targeting PV neurons showed little short-term dynamics. Our results largely agree with in vitro measurements. We therefore demonstrate the technical feasibility of assessing functional cell-type-specific synaptic connectivity in vivo, allowing future investigations into context-dependent modulation of synaptic transmission.

INTRODUCTION

Chemical synaptic transmission is fundamental to brain function and forms the major mechanism for rapid signaling between neurons. Action potentials (APs) evoke calcium influx, driving exocytosis of synaptic vesicles. Fast postsynaptic potentials are evoked by the released neurotransmitter acting upon ionotropic receptors. Early investigations of synaptic transmission in vitro at the frog neuromuscular junction revealed quantal postsynaptic potentials corresponding to release of single synaptic vesicles (Del Castillo and Katz, 1954). The development of in vitro brain slice preparations together with multiple simultaneous intracellular electrophysiological recordings allowed the functional properties of glutamatergic synaptic connectivity and synaptic transmission to be studied in detail between identified pre- and postsynaptic neurons of the mammalian neocortex (Buhl et al., 1997; Reyes et al., 1998; Galarreta and Hestrin, 1998; Beierlein et al., 2003; Holmgren et al., 2003; Koester and Johnston, 2005; Lefort et al., 2009; Hofer et al., 2011; Avermann et al., 2012). These in vitro measurements revealed cell-type-specific synaptic connectivity and cell-type-specific properties of synaptic transmission. Since glutamatergic synapses provide the major excitatory drive for neocortical circuits, these in vitro measurements of glutamatergic synaptic connectivity and synaptic transmission are of fundamental importance for understanding network function. However, due to differences in concentrations of ions, neurotransmitters, neuromodulators, and other molecules, synaptic transmission might be different in vivo. In addition, synaptic connectivity might differ since axonal and dendritic arborisations are truncated by slicing procedures for in vitro recordings. It is therefore of fundamental importance to measure synaptic connectivity and synaptic transmission in vivo.

Few studies have directly investigated synaptic transmission between identified neocortical neurons in vivo, presumably due to the technical difficulties in obtaining intracellular recordings from connected pairs of neurons in vivo (Matsumura et al., 1996; Crochet et al., 2005; Bruno and Sakmann, 2006; Yu and Ferster, 2013). Moreover, it is unknown how synaptic transmission differs among specific neocortical cell types in vivo. Here, we develop a robust technical approach for measuring synaptic transmission between identified neurons in vivo and apply it to investigate excitatory synaptic transmission between single identified layer 2/3 (L2/3) excitatory neurons and two different types of genetically defined postsynaptic GABAergic neurons.

RESULTS

To investigate excitatory synaptic transmission in vivo, we combined optogenetic control of a single excitatory presynaptic neuron with simultaneous whole-cell membrane potential (V_m) recordings to measure unitary excitatory postsynaptic potentials (uEPSPs) in identified GABAergic neurons in L2/3 barrel cortex of the anesthetized mouse (Figure 1A). We delivered plasmid DNA encoding a fast variant of channelrhodopsin-2 (ChR2) (Berndt et al., 2011) and eGFP to a single L2/3 neuron using two-photon guided electroporation (Movie S1, available online) (Kitamura et al., 2008). After 1 day, eGFP expression level was

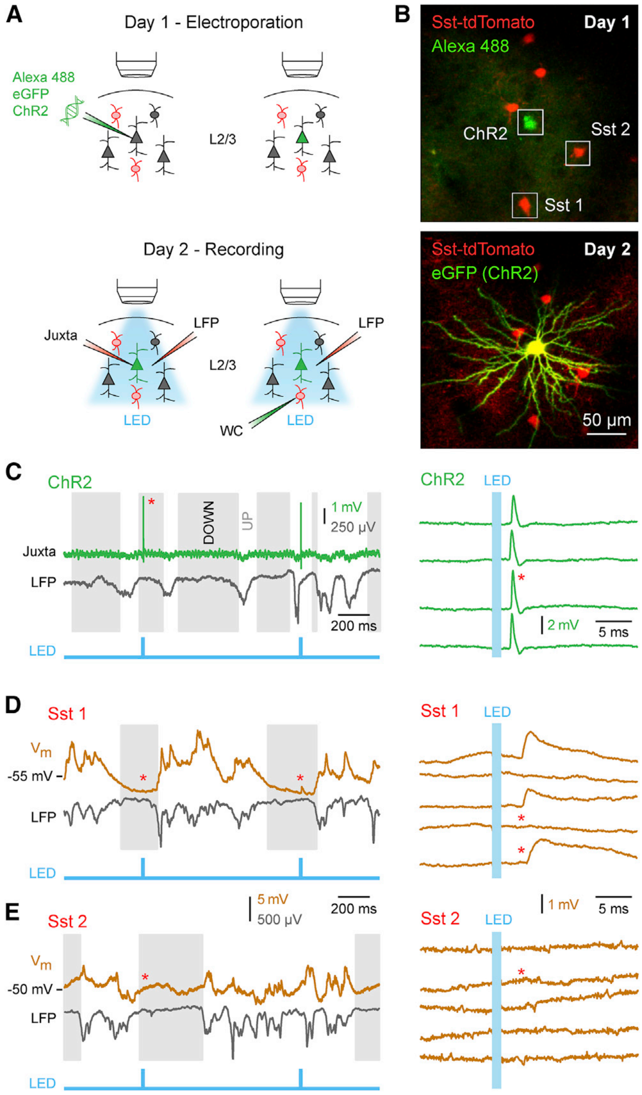

Figure 1. In Vivo Measurement of uEPSPs

(A) On day 1, eGFP- and ChR2-encoding plasmid DNAs together with Alexa 488 dye are electroporated into a single excitatory neuron in L2/3 mouse barrel cortex. On day 2, juxtacellular recording of the ChR2-expressing excitatory neuron is carried out to assess optogenetic control of AP firing. Whole-cell (WC) recordings of nearby tdTomato-expressing neurons are then performed sequentially to measure synaptic potentials. Local field potential (LFP) is recorded simultaneously.

(B) Example in vivo two-photon images of a single L2/3 excitatory neuron filled with Alexa 488 dye in a Sst-Cre × LSL-tdTomato mouse taken immediately after electroporation (above) and 24 hr later showing eGFP expression in soma and dendrites (below).

(C) Juxtacellular recording of the AP firing response to a single 1 ms light pulse delivered at 1 Hz to the ChR2-expressing neuron in (B). LFP recording allowed identification of DOWN (gray) and UP states (white) (left). A single AP was elicited with precise timing by each light pulse during DOWN states (right).

(D) Whole-cell recording of a synaptically connected neuron, Sst 1 in (B), with simultaneous LFP recording (left). Example single-trial uEPSPs and synaptic failures recorded during DOWN states (right).

(E) Same as (D), but for an unconnected Sst neuron, Sst 2 in (B).

See also Movie S1.

sufficiently high to allow morphological validation of the excitatory nature of the electroporated neuron (Figure 1B). In every experiment, we first measured the reliability and temporal precision of the optogenetically evoked presynaptic APs through targeted juxtacellular recording of the ChR2-expressing neuron (Figure 1C). Simultaneous recording of the local field potential (LFP) allowed us to distinguish periods of neuronal network quiescence (DOWN states) from periods of spontaneous depolarization and activity (UP states) (Steriade et al., 1993; Cowan and Wilson, 1994). We then recorded the V_m response to optogenetic single-cell stimulation in genetically defined GABAergic neurons expressing the fluorescent protein tdTomato. In some postsynaptic V_m recordings we observed optogenetically evoked uEPSPs, defining a synaptically connected pair of neurons (Figure 1D). On the other hand, no uEPSPs were detected in V_m recordings from other cells, defining unconnected pairs of neurons (Figure 1E).

Reliable and Precise Optogenetic Control of Action Potential Firing

Quantification of synaptic connectivity and the properties of uEPSPs requires reliable and precise generation of single APs in single identified presynaptic neurons. We therefore measured the reliability and temporal precision of the APs evoked in single ChR2-expressing neurons by optogenetic stimulation in vivo.

We first analyzed APs evoked during the hyperpolarized quiescent DOWN state of the neocortex (Figures 2A and 2B). We delivered single 1 ms blue light flashes at 1 Hz and found that single APs could be evoked reliably (98% ± 10%, n = 44) with a short latency (2.9 ± 1.0 ms) and a low jitter (0.4 ± 0.5 ms) relative to the onset of the blue light flash. We next examined the ability of the optogenetic stimulus to drive high-frequency trains of APs. Using the same light intensity used for evoking single APs, we delivered trains of five 1 ms blue light flashes at 20 Hz (Figure S1) and 50 Hz (Figures 2C and 2D). At these high frequencies, APs could be elicited with equally high probability (20 Hz 100% ± 0%, n = 17; 50 Hz 100% ± 0%, n = 23), short latency (20 Hz 3.2 ± 0.7 ms; 50 Hz 2.5 ± 0.4 ms), and low jitter (20 Hz 0.2 ± 0.1 ms; 50 Hz 0.3 ± 0.1 ms).

Our recording sessions typically lasted ~4.5 hr, and it was therefore important to test the stability of the optogenetic stimulation over long time scales. In a subset of experiments (n = 7), we recorded the APs elicited in the ChR2-expressing neuron at both the beginning and the end of the recording session, delivering the same light stimuli in both cases (Figure 2E). Over this time period, we found that the high probability of evoking APs in response to a single light flash was unchanged (0 hr, 100% ± 0%; 4.5 hr, 100% ± 0%; p = 1), while AP latency (0 hr, 3.4 ± 0.8 ms; 4.5 hr, 2.8 ± 0.7 ms; p = 0.02) and jitter (0 hr, 0.5 ± 0.3 ms; 4.5 hr, 0.2 ± 0.0 ms; p = 0.02) decreased. Similarly, high-frequency optogenetic stimulation was stable in terms of AP probability but also showed shorter AP latency and reduced jitter over ~4.5 hr, which could result from gradually increasing expression levels of ChR2 over the duration of the experiment.

In addition, we examined the impact of spontaneous activity upon the reliability and timing of optogenetically evoked APs (Figures 2F and 2G). We found an equally high light-evoked AP probability in UP states (99% ± 4%) compared to DOWN states (99% ± 7%, p = 1, n = 24), with a slightly higher AP jitter (UP

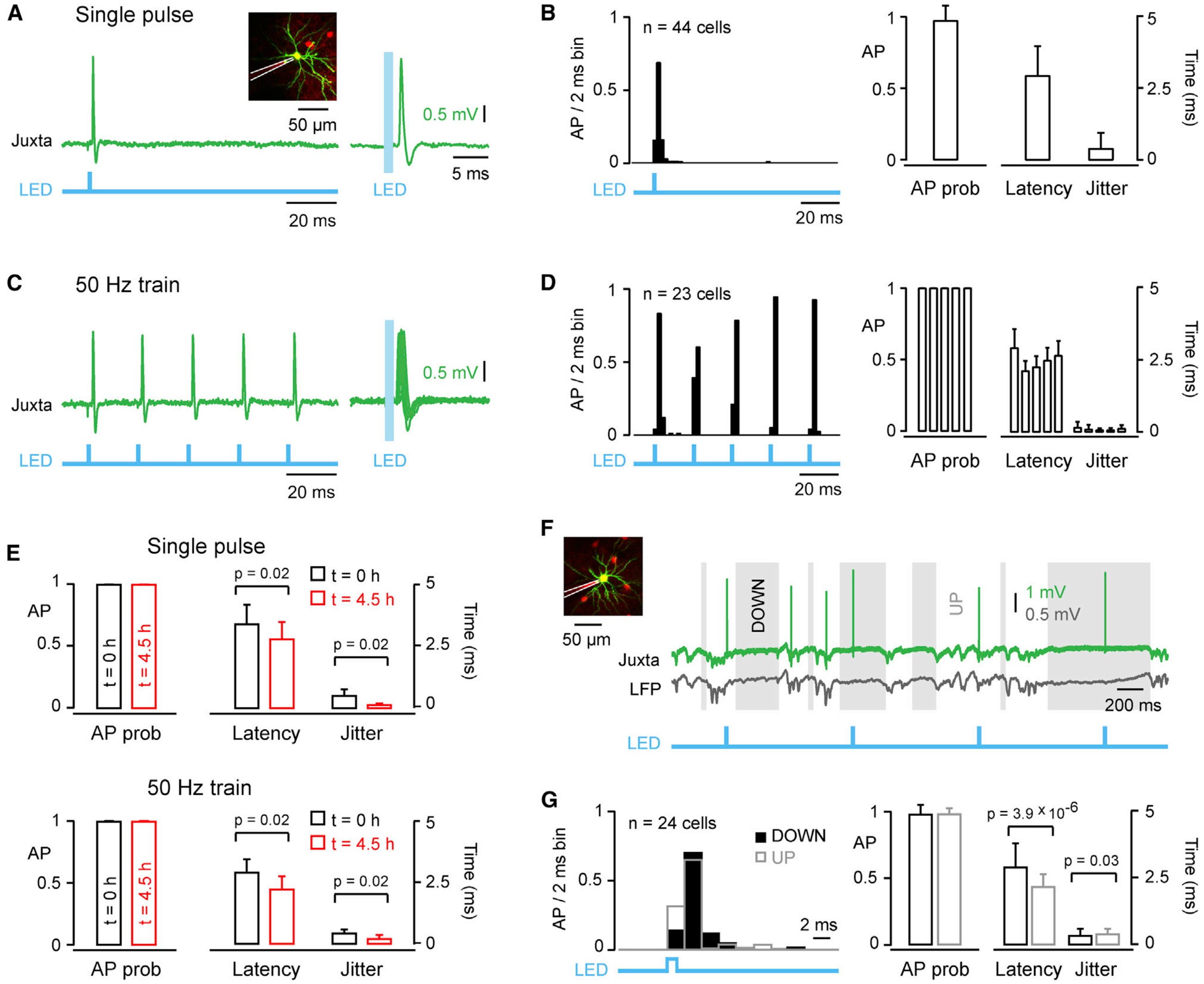

Figure 2. Precise Optogenetic Stimulation of Action Potential Firing in Single Excitatory Neurons In Vivo

(A) Example single AP elicited by a single 1 ms light pulse recorded juxtacellularly in a L2/3 ChR2-expressing excitatory neuron.

(B) Population peristimulus time histogram of light-evoked AP timing (left) and light-evoked AP probability, latency, and jitter (right) for single 1 ms light pulses delivered during the DOWN states.

(C) Same cell as in (A), but for an optogenetic stimulus made of a 50 Hz train of five 1 ms light pulses.

(D) Same analysis as in (B), but for an optogenetic stimulus made of a 50 Hz train of five 1 ms light pulses.

(E) Light-evoked AP probability, latency, and jitter quantified at the beginning (black, t = 0 hr) and end (red, t = 4.5 hr) of the recording session for single 1 ms light pulses (above) and 50 Hz trains of five 1 ms light pulses (below).

(F) Example APs elicited by a single 1 ms light pulse delivered at 1 Hz recorded juxtacellularly during UP and DOWN states.

(G) Population peristimulus time histogram of light-evoked AP timing (left) and light-evoked AP probability, latency, and jitter (right) for 1 ms optogenetic stimuli occurring in DOWN (black) and UP states (gray).

Data are represented as mean ± SD. Two-tail Wilcoxon signed-rank test assessed statistical significance. See also Figure S1.

0.43 ± 0.19 ms; DOWN 0.37 ± 0.25 ms; p = 0.03) and shorter latency (UP 2.2 ± 0.5 ms; DOWN 2.9 ± 0.9 ms; p = 3.9 × 10^{-6}) in UP states compared to DOWN states (Mateo et al., 2011).

In summary, single-cell electroporation of a fast variant of ChR2 allows precise and reliable APs to be optogenetically evoked in L2/3 pyramidal neurons by 1 ms blue light flashes at 1 Hz, 20 Hz, and 50 Hz over many hours during periods of both spontaneous network quiescence and activity, therefore making single-cell optogenetic stimulation well suited for studying uEPSPs.

Unitary Excitatory Synaptic Inputs onto Parvalbumin- and Somatostatin-Expressing GABAergic Neurons

Using two-photon microscopy we targeted whole-cell recordings to parvalbumin-expressing (PV) GABAergic neurons (n = 45; identified in PV-Cre × LSL-tdTomato mice) and somatostatin-expressing (Sst) GABAergic neurons (n = 59; identified in Sst-Cre × LSL-tdTomato mice) (Figure S2). Input resistance (PV 47 ± 22 MΩ; Sst 203 ± 45 MΩ; p = 1.9 × 10^{-16}) and membrane time constant (Tau) (PV 3.6 ± 2.5 ms; Sst 17.7 ± 6.4 ms;

$p = 1.1 \times 10^{-14}$) were larger in Sst compared to PV neurons (Figure S3 and Table S1). AP half-width was smaller in PV than Sst neurons, but AP threshold was similar in both cell types (Figure S3 and Table S1). Mean V_m was more depolarized in Sst compared to PV neurons (PV −66.1 ± 6.0 mV; Sst −59.9 ± 5.4 mV; $p = 3.4 \times 10^{-8}$), while the spontaneous AP rate of PV neurons was higher than that of Sst neurons (PV 5.1 ± 4.1 Hz; Sst 1.0 ± 1.6 Hz; $p = 6.2 \times 10^{-10}$) (Figure S3 and Table S1). The amplitude of slow (1–5 Hz) V_m fluctuations was smaller in Sst neurons compared to PV neurons, and slow V_m oscillations were highly correlated to the local field potential (LFP) for PV neurons but less correlated for Sst neurons (Figure S3 and Table S1). These two types of GABAergic neurons therefore have diverse intrinsic electrophysiological features in vivo, and their distinct patterns of spontaneous membrane potential fluctuations might be driven by different synaptic input.

By optogenetically stimulating the presynaptic ChR2-expressing excitatory neuron, we assessed the excitatory synaptic connectivity onto these two types of GABAergic neurons during the DOWN state (Figure 3A). The connection probability between excitatory and PV neurons (51%; connected/tested, 23/45) was significantly higher (p = 0.03) than the connection probability between excitatory and Sst neurons (31%; connected/tested, 18/59) (Figure 3B). Within the small range of distances explored (<125 μm), we did not find a correlation of the synaptic connectivity with respect to the distance separating the somata of the presynaptic and the postsynaptic neurons (PV r^2 = 0.19, p = 0.56; Sst r^2 = 0.01, p = 0.89) (Figure 3C).

The distribution of uEPSP amplitudes during the DOWN state in PV and Sst neurons was different (PV median 0.39 mV; Sst median 0.21 mV; p = 0.03), although means were similar (PV 0.53 ± 0.39 mV; Sst 0.50 ± 0.86 mV (Figure 3D and Table S2). The failure rate of synaptic transmission was lower in PV neurons compared to Sst neurons (PV 27% ± 16%; Sst 68% ± 30%; p = 0.0001) and inversely related to uEPSP amplitude in both neuron types (PV $\rho = -0.79$, $p = 2.1 \times 10^{-5}$; Sst $\rho = -0.83$, $p = 6.6 \times 10^{-5}$) (Figure 3E and Table S2). Similarly, the coefficient of variation of uEPSP amplitude was smaller in PV neurons compared to Sst neurons (PV 0.33 ± 0.28; Sst 0.92 ± 0.53; $p = 6.1 \times 10^{-4}$) (Figure 3F and Table S2).

The time course of uEPSPs also differed strongly between PV and Sst neurons. The 20%–80% rise time of uEPSPs was faster in PV than in Sst neurons (PV 0.68 ± 0.32 ms; Sst 1.76 ± 1.40 ms; $p = 8.2 \times 10^{-6}$). The half-width duration of uEPSPs was shorter in PV than Sst neurons (PV 4.0 ± 1.4 ms; Sst 11.6 ± 6.7 ms; $p = 2.1 \times 10^{-5}$), as was the exponential time constant of the decaying phase of the uEPSPs (PV 5.2 ± 3.0 ms; Sst 16.0 ± 8.5 ms; $p = 2.7 \times 10^{-5}$) (Figure 3G and Table S2).

Finally, we compared uEPSPs evoked during UP and DOWN states (Figure 3H). Although there were significant decreases in uEPSP amplitude in 5 out of 11 PV neurons and 1 out of 6 Sst neurons during UP states, overall we found that uEPSP amplitude was similar across states in both PV neurons (UP 0.41± 0.42 mV; DOWN 0.48 ± 0.33 mV; p = 0.32, n = 11) and Sst neurons (UP 0.38 ± 0.36 mV; DOWN 0.32 ± 0.42 mV; p = 0.56, n = 6) (Figure 3I). Baseline V_m at uEPSP onset was different between the two network states in both PV (UP −49.9 ± 1.9 mV; DOWN −66.0 ± 2.1 mV; $p = 9.8 \times 10^{-4}$) and Sst neurons (UP −57.0 ± 5.7 mV; DOWN −62.0 ± 8.2 mV; p = 0.03) (Figure 3J).

Short-Term Synaptic Dynamics

The temporal pattern of presynaptic AP firing strongly influences excitatory synaptic transmission. We therefore measured in vivo uEPSP dynamics evoked by stimulating the presynaptic excitatory ChR2 neuron to fire a burst of five APs at 20 Hz (Figure S4) or 50 Hz (Figure 4A). At a stimulation frequency of 50 Hz, synapses targeting Sst neurons showed strong facilitation, whereas excitatory input to PV neurons showed a relatively reliable response with little short-term dynamics (uEPSP5 to uEPSP1 amplitude ratio: Sst 9.2 ± 5.0; PV 1.0 ± 0.2, mean ± SEM; p = 0.01) (Figures 4B and 4C). uEPSPs elicited in Sst neurons (but not PV neurons) also showed pronounced temporal summation, as measured by the depolarized baseline V_m at the onset of sequential uEPSPs (ΔBaseline V_m for uEPSP5: Sst 1.05 ± 0.28 mV; PV 0.08 ± 0.05 mV, mean ± SEM; $p = 8.7 \times 10^{-5}$) (Figures 4B and 4D).

DISCUSSION

By combining single-cell optogenetics with whole-cell V_m recordings, we systematically and directly quantified excitatory synaptic transmission onto PV- and Sst-expressing GABAergic neurons in L2/3 of the mouse barrel cortex in vivo. We found that PV and Sst neurons exhibit distinct intrinsic electrophysiological properties and receive local excitatory synaptic input with different connectivity, speed, reliability, and short-term dynamics in vivo. Our results extend current knowledge of cell-type-specific neuronal communication in vitro to the intact and spontaneously active neocortex in vivo.

Single-Cell Optogenetics

Measurement of unitary postsynaptic potentials requires single APs to be precisely evoked in single presynaptic neurons. To date, this has been accomplished in electrophysiological recordings by injection of current either intracellularly or extracellularly during juxtacellular recording. Here, we show that single-cell electroporation of ChR2 provides an alternative method for precise stimulation with high reliability and low temporal jitter (Figure 2). Although high levels of ChR2 in axons could enhance calcium entry, thereby increasing neurotransmitter release probability in an unphysiological manner, our in vivo measurements of short-term plasticity rather suggest release probability lower than that expected from previous in vitro measurements using dual whole-cell recordings (see below). The optogenetic approach offers anatomical identification of the presynaptic neuron through expression of fluorescent proteins and allows long-term stimulation of the same neuron, tested here on the time scale of a few hours. The ability to stimulate the same neuron over long periods of time allows synaptic connectivity from the same presynaptic neuron to be assessed onto different potential postsynaptic neurons recorded sequentially (Figure 1). In future studies, it will be interesting to apply single-cell optogenetic stimulation paradigms to study behavioral effects of single-cell stimulation, which have so far been hampered by the short durations typically associated with intracellular and juxtacellular recordings in behaving animals (Houweling and Brecht, 2008).

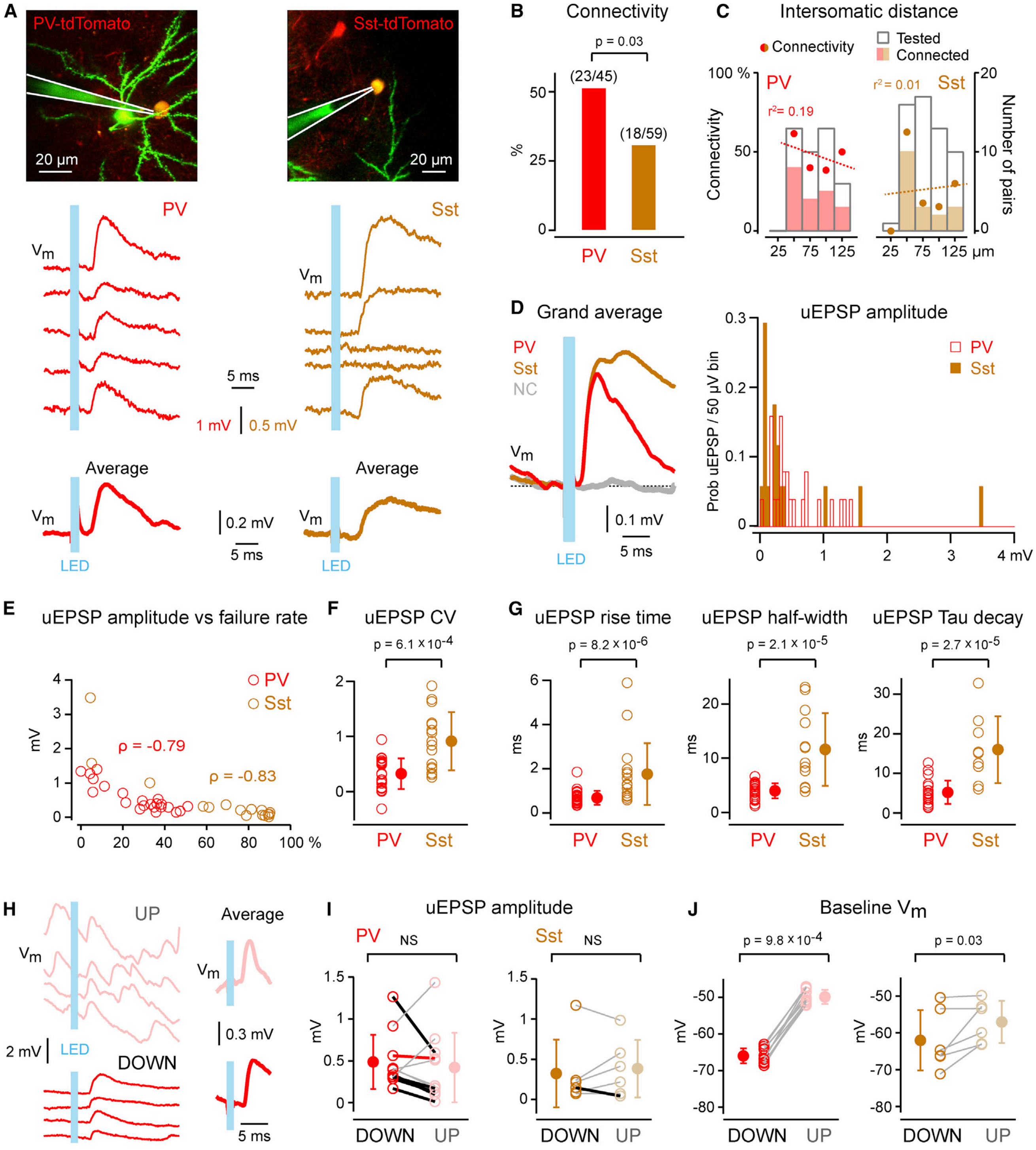

Figure 3. Cell-Type-Specific Features of Excitatory Synaptic Transmission In Vivo

(A) Example whole-cell recording of uEPSPs elicited in a PV neuron (red) and a Sst neuron (brown) during DOWN states by 1 ms light pulses. Single trial uEPSPs are shown above and average uEPSP below. The in vivo two-photon images show the whole-cell recording pipette (Alexa 488 dye, green), the recorded tdTomato-expressing neuron (yellow), and part of the presynaptic eGFP- and ChR2-expressing neuron (green).

(B) Connectivity rate is higher from excitatory neurons onto PV neurons than onto Sst neurons.

(C) Connectivity rate is uncorrelated with intersomatic distance for both Exc→PV (p = 0.56) (left) and Exc→Sst pairs (p = 0.89) (right) over the short range tested.

(D) uEPSP grand average of all connected PV and Sst neurons, as well as that of all nonconnected (NC) neurons (gray) (left) and uEPSP amplitude distribution (right). The uEPSP amplitude for each cell was computed as the average across both failure and success trials.

(legend continued on next page)

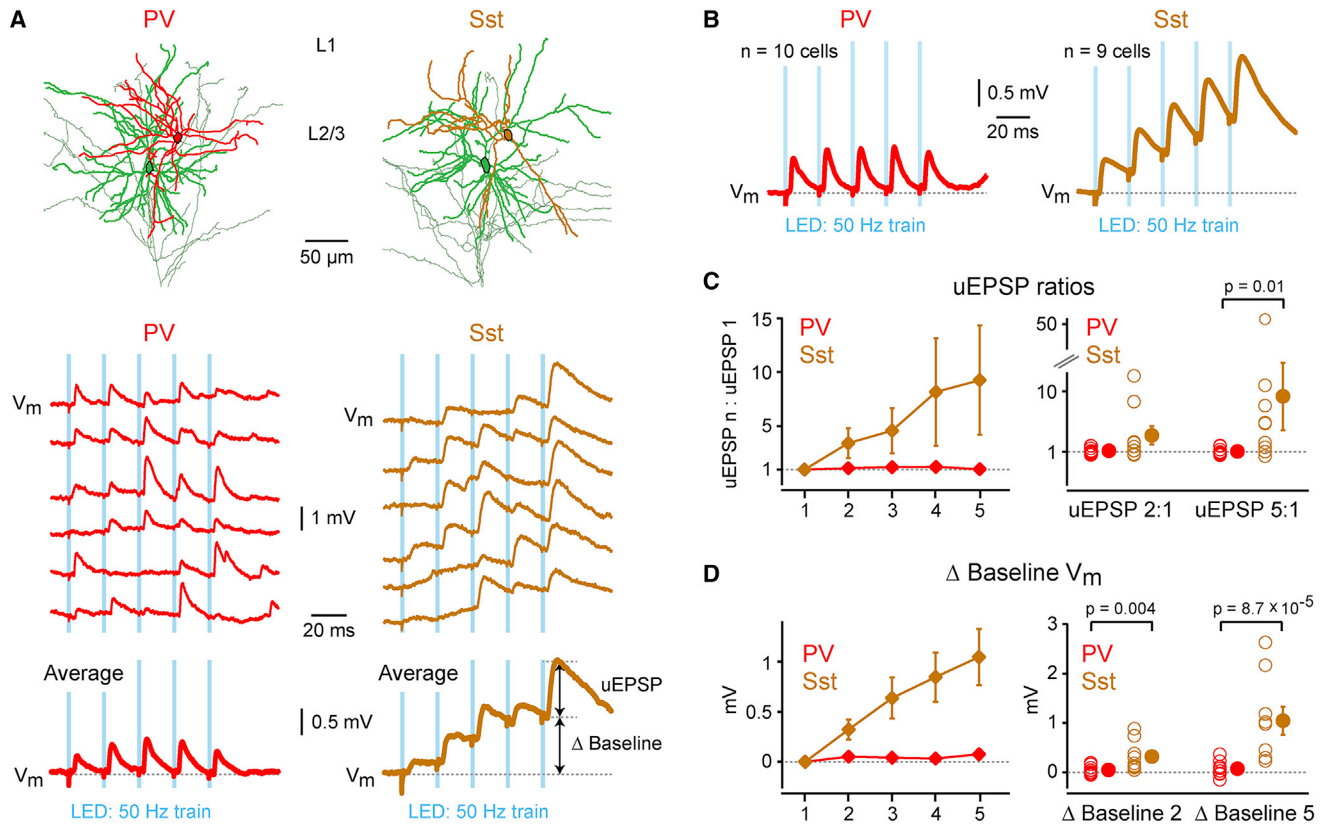

Figure 4. In Vivo Short-Term Synaptic Dynamics

(A) Reconstruction of connected pairs of L2/3 Exc→PV and Exc→Sst neurons. Dendrites of the presynaptic excitatory neurons are colored in green, axons in gray. Dendrites of postsynaptic PV and Sst neurons are colored in red and brown, respectively. Example whole-cell recording of uEPSPs elicited in the PV (red) and Sst (brown) neuron during DOWN states by a 50 Hz train of five 1 ms light pulses. Single trial uEPSPs are shown above and average uEPSPs below.

(B) Grand average uEPSPs for all connected PV and Sst neurons evoked by 50 Hz train of optogenetic stimuli during DOWN states.

(C) Population uEPSP amplitude ratios comparing the amplitude of each uEPSP in the train to the amplitude of the first uEPSP for PV and Sst neurons (left). Individual neuron uEPSP amplitude ratios for uEPSP2 and uEPSP5 (right). Exc→Sst synapses facilitate, whereas Exc→PV synapses show little short-term dynamics.

(D) Population difference in baseline V_m of each uEPSP in the train relative to the baseline V_m of the first uEPSP for PV and Sst neurons (left). Differences across individual neurons in baseline V_m at onset of uEPSP2 and uEPSP5 (right). uEPSPs summate prominently in Sst neurons, but not in PV neurons.

Data are represented as mean ± SEM. Two-tail Wilcoxon rank-sum test assessed statistical significance. See also Figure S4.

In Vivo versus In Vitro Measurements of Synaptic Connectivity

Cell-type-specific measurements of synaptic connectivity in the neocortex have so far been carried out in vitro in brain slice preparations. Axonal and dendritic arborisations are typically truncated during the preparation of brain slices, which could reduce the apparent measured synaptic connectivity. Here, we found that excitatory L2/3 pyramidal neurons in mouse barrel cortex

(E) uEPSP amplitude is anticorrelated with the failure rate for both Exc→PV and Exc→Sst synapses.

(F) uEPSP coefficient of variation (CV) is larger for Sst neurons compared to PV neurons.

(G) uEPSP 20%–80% rise time, full-width at half-maximum amplitude, and exponential decay time constant (Tau) are slower for Sst neurons compared to PV neurons.

(H) Example whole-cell recording of uEPSPs elicited in a PV neuron during DOWN (below) and UP states (above) by 1 ms light pulses. Single trial uEPSPs are shown on the left and average uEPSPs on the right.

(I) uEPSPs elicited in DOWN states on average have an amplitude similar to that of uEPSPs elicited in UP states for both PV and Sst neurons (left). One Sst and five PV neurons show a significant decrease in uEPSP amplitude in UP compared to DOWN states (black lines). Red line represents neuron in (H).

(J) Baseline V_m at uEPSP onset is more depolarized in UP compared to DOWN states for both PV and Sst neurons (right).

Data are represented as mean ± SD. χ^2 test assessed for statistical difference in connectivity rates. Linear regression tested distance dependence of connectivity. Two-tail Wilcoxon rank-sum test assessed the difference in uEPSP CV, rise time, half-width, and Tau decay. Two-tail Wilcoxon signed-rank test assessed the differences in uEPSP amplitude and baseline V_m between UP and DOWN states. Spearman's ρ assessed the correlation between uEPSP amplitude and failure rate. See also Figures S2 and S3 and Tables S1 and S2.

in vivo provide synaptic input onto 51% (23/45) of nearby PV neurons (Figure 3). Closely related in vitro measurements from L2/3 barrel cortex found a similar connectivity of excitatory to PV neurons: mouse 58% (23/40) (Avermann et al., 2012) and rat 48% (19/40) (Kapfer et al., 2007). There is general agreement that synaptic connectivity is high from excitatory to PV cells (Holmgren et al., 2003; Hofer et al., 2011). The in vitro connectivity of excitatory and Sst neurons in rat L2/3 barrel cortex was determined to be 29% (Kapfer et al., 2007), in good agreement with our in vivo measurements of 31% (18/59) (Figure 3). However, there are also reports of higher levels of connectivity from excitatory to Sst L2/3 cells (Fanselow and Connors, 2010), and in rat L4 barrel cortex excitatory neurons were even found to connect preferentially to Sst compared to PV neurons (Beierlein et al., 2003). In addition to differences across cortical layers, it is also likely that synaptic connectivity will vary across cortical regions (Yoshimura and Callaway, 2005; Levy and Reyes, 2012).

Properties of uEPSPs in PV and Sst Neurons Measured In Vivo

The uEPSPs measured in PV and Sst neurons had markedly different properties. On a trial-by-trial basis, the amplitude of uEPSPs had low variance and low failure rate in PV neurons, whereas the uEPSPs in Sst neurons had high variance and high failure rate. This suggests that the probability of releasing synaptic vesicles in response to an AP is lower for synapses onto Sst neurons (Buhl et al., 1997; Koester and Johnston, 2005). The clear distinction of failure and success trials in postsynaptic Sst neurons (Figures 1 and 3) presumably results from the very high input resistance of the Sst neurons (~200 MΩ). The unreliable synaptic input to Sst neurons may contribute to the low correlation of V_m fluctuations in Sst neurons with the LFP, whereas PV neurons receive more reliable input from nearby excitatory neurons, thus giving high correlations with the LFP (Figure S3H). Differences in the properties of excitatory synaptic transmission might therefore contribute to the different V_m correlations of PV, Sst, and excitatory neurons in awake mice (Gentet et al., 2010, 2012).

The time course of the uEPSPs was also very different in PV and Sst neurons. The uEPSP rise time was faster in PV neurons compared to Sst neurons. The uEPSP duration was also much longer in Sst neurons compared to PV neurons. The different kinetics of the uEPSPs likely result from the intrinsic electrophysiological properties of the membrane time constants. PV neurons had a uEPSP decay time of 5.2 ms and a membrane time constant of 3.6 ms, whereas Sst neurons had a uEPSP decay time of 16.0 ms and a membrane time constant of 17.7 ms (Figure 3 and Tables S1 and S2). PV and Sst neurons have very little synaptic NMDA conductance (Matta et al., 2013), and excitation is therefore largely mediated by AMPA receptors, which typically evoke very brief synaptic conductances (~2 ms). The membrane time constant therefore contributes importantly to the duration of the uEPSP measured at the soma.

PV neurons therefore appear to be designed for reliable and rapid signal processing, receiving brief, fast-rising uEPSPs with a low failure rate. In contrast, Sst neurons receive unreliable excitatory input and process it over much longer time scales, having long membrane time constants and therefore long-duration uEPSPs, which thus promote summation of uEPSPs (Figure 4D).

Short-Term Synaptic Plasticity

We found that uEPSPs recorded in Sst neurons facilitated strongly in response to high-frequency stimulation of the presynaptic neuron (Figures 4 and S4). Our in vivo measurements are in good agreement with previous in vitro measurements showing strong short-term facilitation in postsynaptic Sst neurons (Reyes et al., 1998; Rozov et al., 2001; Beierlein et al., 2003; Koester and Johnston, 2005; Silberberg and Markram, 2007; Kapfer et al., 2007; Fanselow and Connors, 2010). The facilitation presumably results from the low release probability observed under baseline low-frequency stimulation, which allows for strong increases in release probability as calcium summates in the presynaptic boutons during high-frequency stimulation.

On the other hand, the reliable uEPSPs exhibiting little short-term plasticity in PV neurons that we found in vivo contrasts with the strongly depressing synaptic input typically reported for these neurons in vitro (Reyes et al., 1998; Rozov et al., 2001; Galarreta and Hestrin, 1998; Holmgren et al., 2003; Koester and Johnston, 2005; Kapfer et al., 2007; Hofer et al., 2011). Interestingly, direct comparison of synaptic transmission in vitro and in vivo at the calyx of Held also showed less synaptic depression in vivo due to elevated presynaptic firing rates in vivo, elevated neurotransmitter concentrations in vivo, and lower extracellular calcium concentrations in vivo compared to the typical values used in slice experiments (Lorteije et al., 2009).

Synaptic Transmission across Cortical States—Future Perspectives

Although on average we did not find a consistent modulation of uEPSPs in PV or Sst neurons comparing quiescent cortical states (DOWN) and active cortical states (UP) (Figure 3H), in a few cells we found that uEPSP amplitude decreased significantly during UP states. Decreases in uEPSP amplitude during UP states (Crochet et al., 2005; Bruno and Sakmann, 2006) would be expected because the electrical driving force is different, with UP states being depolarized compared to DOWN states. In addition, the synaptic input occurring during UP states causes decreases in input resistance in some experimental preparations (Destexhe et al., 2003), but not others (Waters and Helmchen, 2006; Mateo et al., 2011). On the other hand, depolarization can also enhance presynaptic neurotransmitter release (Shu et al., 2006) and activate postsynaptic voltage-gated somatic and dendritic conductances, which could boost uEPSP amplitude. The regulation of synaptic transmission across cortical states may therefore be complicated and deserves further detailed investigation. It is also possible that anesthesia directly affects synaptic transmission. In future experiments, it will therefore be important to extend these first in vivo measurements of cell-type-specific synaptic transmission to other well-defined neocortical cell types and to compare synaptic transmission across different behavioral states in awake mice.

EXPERIMENTAL PROCEDURES

All experiments were carried out in accordance with protocols approved by the Swiss Federal Veterinary Office (see Supplemental Experimental Procedures).

SUPPLEMENTAL INFORMATION

Supplemental Information includes Supplemental Experimental Procedures, four figures, two tables, and one movie and can be found with this article online at http://dx.doi.org/10.1016/j.neuron.2014.11.025.

ACKNOWLEDGMENTS

We thank Thomas Oertner for plasmid DNA encoding the E123T/T159C variant of ChR2, James Cottam and Benjamin Judkewitz from Michael Häusser's lab for help with electroporation, and Julien Duc for help with data analysis. We thank Sylvain Crochet, Takayuki Yamashita, Yves Kremer, Céline Mateo, Luc Gentet, Tanya Sippy, and Aaron Clark for helpful discussions. This work was funded by a grant from the Swiss National Competence Center for Biomedical Imaging, the Swiss National Science Foundation, and the European Research Council.

Accepted: November 20, 2014
Published: December 24, 2014

REFERENCES

Avermann, M., Tomm, C., Mateo, C., Gerstner, W., and Petersen, C.C.H. (2012). Microcircuits of excitatory and inhibitory neurons in layer 2/3 of mouse barrel cortex. J. Neurophysiol. *107*, 3116–3134.

Beierlein, M., Gibson, J.R., and Connors, B.W. (2003). Two dynamically distinct inhibitory networks in layer 4 of the neocortex. J. Neurophysiol. *90*, 2987–3000.

Berndt, A., Schoenenberger, P., Mattis, J., Tye, K.M., Deisseroth, K., Hegemann, P., and Oertner, T.G. (2011). High-efficiency channelrhodopsins for fast neuronal stimulation at low light levels. Proc. Natl. Acad. Sci. USA *108*, 7595–7600.

Bruno, R.M., and Sakmann, B. (2006). Cortex is driven by weak but synchronously active thalamocortical synapses. Science *312*, 1622–1627.

Buhl, E.H., Tamás, G., Szilágyi, T., Stricker, C., Paulsen, O., and Somogyi, P. (1997). Effect, number and location of synapses made by single pyramidal cells onto aspiny interneurones of cat visual cortex. J. Physiol. *500*, 689–713.

Cowan, R.L., and Wilson, C.J. (1994). Spontaneous firing patterns and axonal projections of single corticostriatal neurons in the rat medial agranular cortex. J. Neurophysiol. *71*, 17–32.

Crochet, S., Chauvette, S., Boucetta, S., and Timofeev, I. (2005). Modulation of synaptic transmission in neocortex by network activities. Eur. J. Neurosci. *21*, 1030–1044.

Del Castillo, J., and Katz, B. (1954). Quantal components of the end-plate potential. J. Physiol. *124*, 560–573.

Destexhe, A., Rudolph, M., and Paré, D. (2003). The high-conductance state of neocortical neurons in vivo. Nat. Rev. Neurosci. *4*, 739–751.

Fanselow, E.E., and Connors, B.W. (2010). The roles of somatostatin-expressing (GIN) and fast-spiking inhibitory interneurons in UP-DOWN states of mouse neocortex. J. Neurophysiol. *104*, 596–606.

Galarreta, M., and Hestrin, S. (1998). Frequency-dependent synaptic depression and the balance of excitation and inhibition in the neocortex. Nat. Neurosci. *1*, 587–594.

Gentet, L.J., Avermann, M., Matyas, F., Staiger, J.F., and Petersen, C.C.H. (2010). Membrane potential dynamics of GABAergic neurons in the barrel cortex of behaving mice. Neuron *65*, 422–435.

Gentet, L.J., Kremer, Y., Taniguchi, H., Huang, Z.J., Staiger, J.F., and Petersen, C.C.H. (2012). Unique functional properties of somatostatin-expressing GABAergic neurons in mouse barrel cortex. Nat. Neurosci. *15*, 607–612.

Hofer, S.B., Ko, H., Pichler, B., Vogelstein, J., Ros, H., Zeng, H., Lein, E., Lesica, N.A., and Mrsic-Flogel, T.D. (2011). Differential connectivity and response dynamics of excitatory and inhibitory neurons in visual cortex. Nat. Neurosci. *14*, 1045–1052.

Holmgren, C., Harkany, T., Svennenfors, B., and Zilberter, Y. (2003). Pyramidal cell communication within local networks in layer 2/3 of rat neocortex. J. Physiol. *551*, 139–153.

Houweling, A.R., and Brecht, M. (2008). Behavioural report of single neuron stimulation in somatosensory cortex. Nature *451*, 65–68.

Kapfer, C., Glickfeld, L.L., Atallah, B.V., and Scanziani, M. (2007). Supralinear increase of recurrent inhibition during sparse activity in the somatosensory cortex. Nat. Neurosci. *10*, 743–753.

Kitamura, K., Judkewitz, B., Kano, M., Denk, W., and Häusser, M. (2008). Targeted patch-clamp recordings and single-cell electroporation of unlabeled neurons in vivo. Nat. Methods *5*, 61–67.

Koester, H.J., and Johnston, D. (2005). Target cell-dependent normalization of transmitter release at neocortical synapses. Science *308*, 863–866.

Lefort, S., Tomm, C., Floyd Sarria, J.C., and Petersen, C.C.H. (2009). The excitatory neuronal network of the C2 barrel column in mouse primary somatosensory cortex. Neuron *61*, 301–316.

Levy, R.B., and Reyes, A.D. (2012). Spatial profile of excitatory and inhibitory synaptic connectivity in mouse primary auditory cortex. J. Neurosci. *32*, 5609–5619.

Lorteije, J.A., Rusu, S.I., Kushmerick, C., and Borst, J.G. (2009). Reliability and precision of the mouse calyx of Held synapse. J. Neurosci. *29*, 13770–13784.

Mateo, C., Avermann, M., Gentet, L.J., Zhang, F., Deisseroth, K., and Petersen, C.C.H. (2011). In vivo optogenetic stimulation of neocortical excitatory neurons drives brain-state-dependent inhibition. Curr. Biol. *21*, 1593–1602.

Matsumura, M., Chen, D., Sawaguchi, T., Kubota, K., and Fetz, E.E. (1996). Synaptic interactions between primate precentral cortex neurons revealed by spike-triggered averaging of intracellular membrane potentials in vivo. J. Neurosci. *16*, 7757–7767.

Matta, J.A., Pelkey, K.A., Craig, M.T., Chittajallu, R., Jeffries, B.W., and McBain, C.J. (2013). Developmental origin dictates interneuron AMPA and NMDA receptor subunit composition and plasticity. Nat. Neurosci. *16*, 1032–1041.

Reyes, A., Lujan, R., Rozov, A., Burnashev, N., Somogyi, P., and Sakmann, B. (1998). Target-cell-specific facilitation and depression in neocortical circuits. Nat. Neurosci. *1*, 279–285.

Rozov, A., Burnashev, N., Sakmann, B., and Neher, E. (2001). Transmitter release modulation by intracellular Ca2+ buffers in facilitating and depressing nerve terminals of pyramidal cells in layer 2/3 of the rat neocortex indicates a target cell-specific difference in presynaptic calcium dynamics. J. Physiol. *531*, 807–826.

Shu, Y., Hasenstaub, A., Duque, A., Yu, Y., and McCormick, D.A. (2006). Modulation of intracortical synaptic potentials by presynaptic somatic membrane potential. Nature *441*, 761–765.

Silberberg, G., and Markram, H. (2007). Disynaptic inhibition between neocortical pyramidal cells mediated by Martinotti cells. Neuron *53*, 735–746.

Steriade, M., Nuñez, A., and Amzica, F. (1993). A novel slow (< 1 Hz) oscillation of neocortical neurons in vivo: depolarizing and hyperpolarizing components. J. Neurosci. *13*, 3252–3265.

Waters, J., and Helmchen, F. (2006). Background synaptic activity is sparse in neocortex. J. Neurosci. *26*, 8267–8277.

Yoshimura, Y., and Callaway, E.M. (2005). Fine-scale specificity of cortical networks depends on inhibitory cell type and connectivity. Nat. Neurosci. *8*, 1552–1559.

Yu, J., and Ferster, D. (2013). Functional coupling from simple to complex cells in the visually driven cortical circuit. J. Neurosci. *33*, 18855–18866.

Neuron, Volume *85*

Supplemental Information

In Vivo Measurement of Cell-Type-Specific Synaptic Connectivity and Synaptic Transmission in

Layer 2/3 Mouse Barrel Cortex

Aurélie Pala and Carl C.H. Petersen

Figure S1

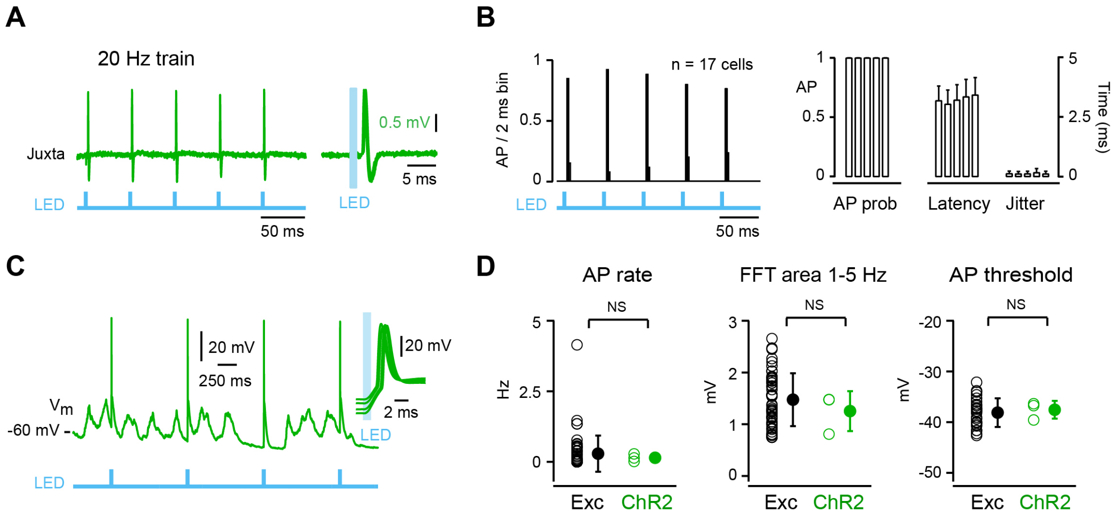

Figure S1. Optogenetic control of action potential firing of a single excitatory neuron in vivo, related to Figure 2.

(A) Example single APs elicited by an optogenetic stimulus made of a 20 Hz train of five 1-ms light pulses recorded juxtacellularly in the same L2/3 ChR2-expressing excitatory neuron as in Figure 2.

(B) Population peristimulus time histogram of light-evoked AP timing *(left)* and light-evoked AP probability, latency and jitter *(right)* for an optogenetic stimulus made of a 20 Hz train of five 1-ms light pulses.

(C) Example whole-cell recording of single APs elicited by single 1-ms light pulses delivered at 1 Hz in a different ChR2-expressing excitatory neuron than in (A). Inset shows magnified APs shape.

(D) Spontaneous AP rate, V_m 1-5 Hz FFT amplitude and AP threshold were similar in ChR2-expressing excitatory neurons (n = 3 cells) compared to non-expressing excitatory neurons.

Data are represented as mean ± SD. Two-tail Wilcoxon rank-sum test assessed statistical significance.

Figure S2

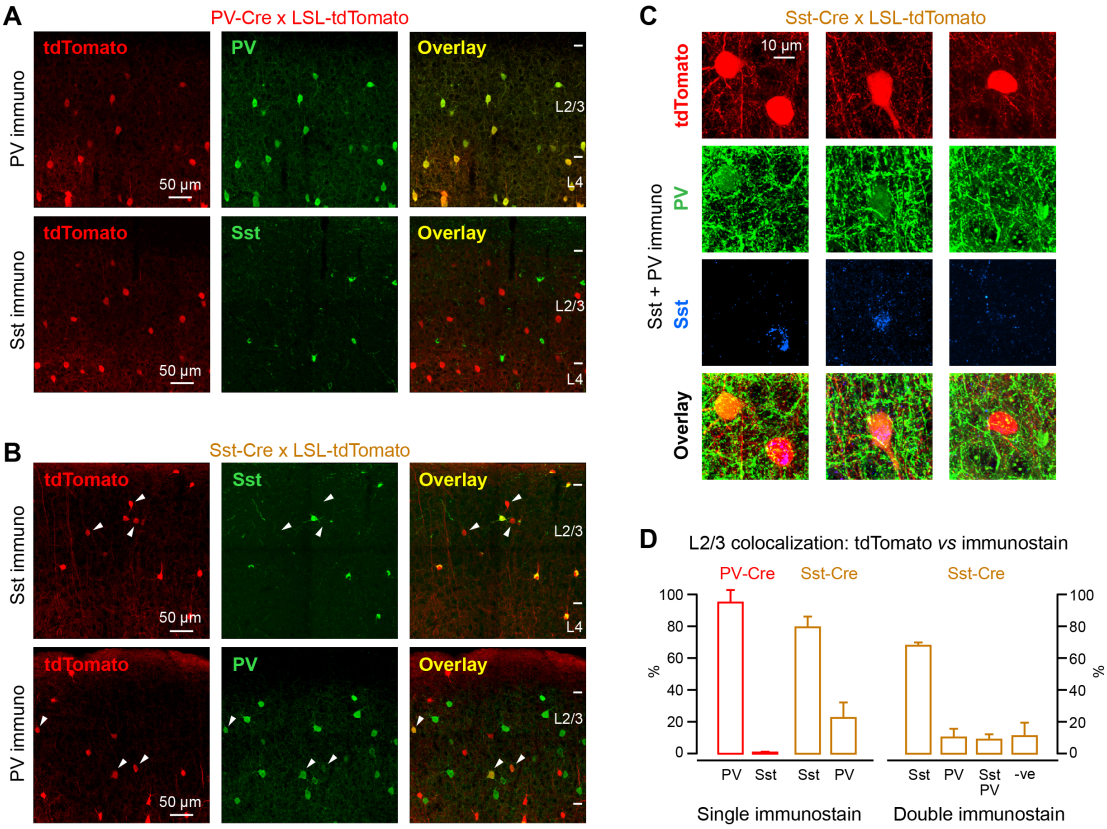

Figure S2. Immunostaining against PV and Sst in barrel cortex of PV-Cre x LSL-tdTomato and Sst-Cre x LSL-tdTomato mice, related to Figure 3.

(A) Example of single immunostaining against PV or Sst (green) in barrel cortex upper layers of a PV-Cre x LSL-tdTomato mouse (red).

(B) Same as in (A) but for a Sst-Cre x LSL-tdTomato mouse. Some L2/3 tdTomato-expressing neurons are not positive for Sst (*above*, arrows) while some L2/3 tdTomato-expressing neurons are positive for PV (*below*, arrows).

(C) Example of double immunostaining against both PV (green) and Sst (blue) in a Sst-Cre x LSL-tdTomato mouse (red). Some L2/3 tdTomato-expressing neurons are positive only for Sst while some are positive only for PV (*left*), some are positive for both Sst and PV (*middle*) and some are negative for both PV and Sst (*right*).

(D) In L2/3 barrel cortex of PV-Cre x LSL-tdTomato mice 95.6 ± 7.6 % (n = 270 cells across 3 mice) of tdTomato-expressing neurons express PV while 0.8 ± 0.7 % (n = 284 cells across 3 mice) express Sst (red, *left*). In L2/3 barrel cortex of Sst-Cre x LSL-tdTomato mouse 80.0 ± 6.5 % (n = 157 cells across 3 mice) of tdTomato-expressing neurons express Sst while 23.0 ± 9.5 % (n = 181 cells across 3 mice) express PV (brown, *left*). Double immunostaining (*right*) shows that 68.4 ± 1.8 % of Sst-tdTomato neurons express Sst only, 10.7 ± 5.3 % express PV only, 9.4 ± 3.1 %

express both Sst and PV, and 11.5 ± 8.2 % express neither PV nor Sst (n = 181 cells across 3 mice).

Data are represented as mean ± SD.

Figure S3

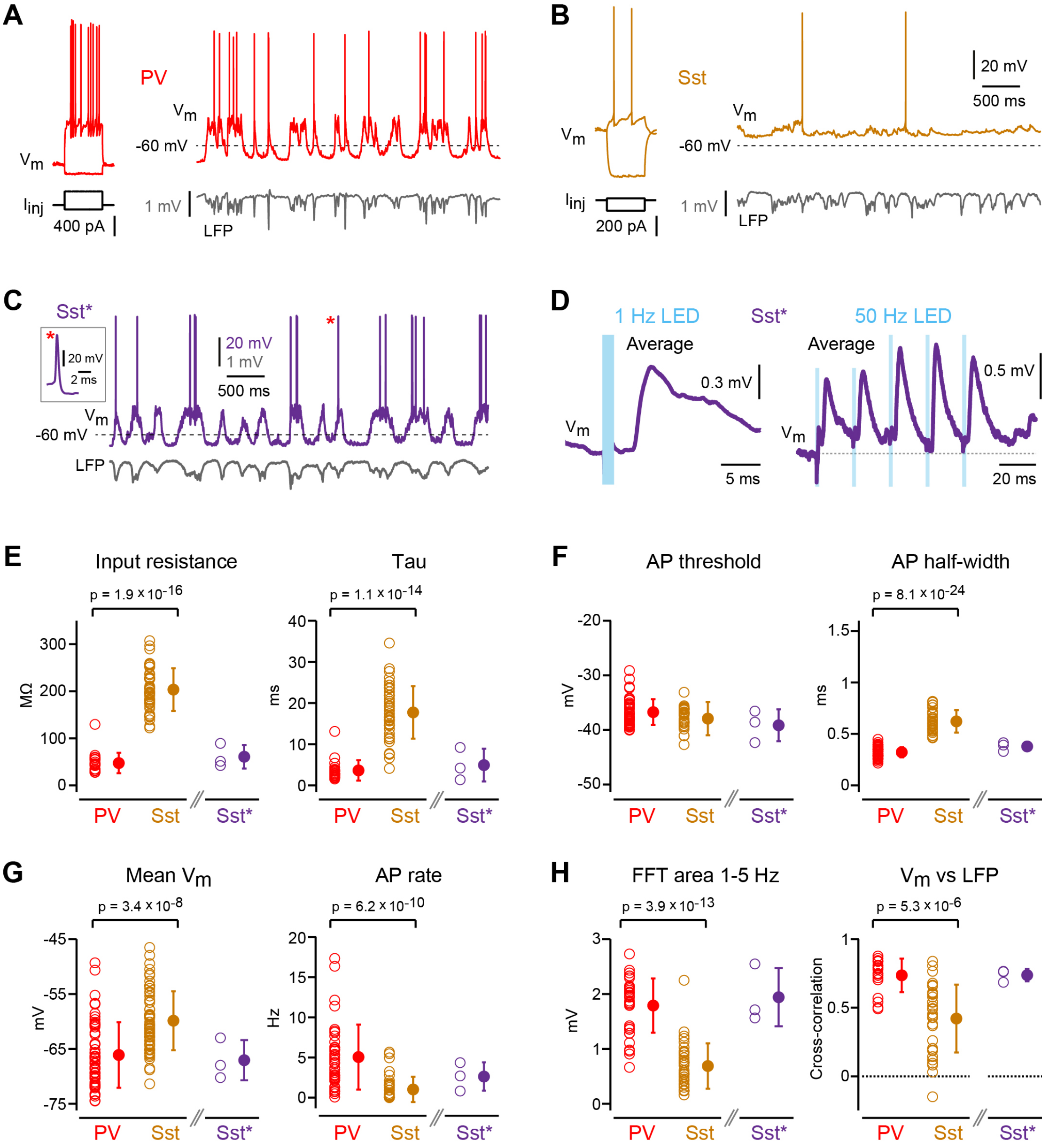

Figure S3. Distinct electrophysiological properties of PV and Sst neurons in L2/3 mouse barrel cortex in vivo, related to Figure 3.

(A) Example whole-cell recording of rheobase AP firing and spontaneous V_m dynamics together with LFP recording for a PV neuron.

(B) Same as in (A) but for a Sst neuron.

(C) A small subset of Sst neurons (Sst*, n = 3 out of 66 recorded Sst-Cre x tdTomato neurons) with distinct electrophysiological properties are excluded from the Sst dataset because they are likely to be PV neurons. Example whole-cell recording of a Sst* neuron. Inset shows magnified AP shape. This Sst* neuron shows large V_m slow-wave oscillation amplitude and relatively high firing rate with a narrow and fast AP waveform, similar to PV neurons.

(D) Average uEPSPs elicited during DOWN state in the same Sst* neuron as in (C) by a 1-ms light pulse *(left)* and by a 50 Hz train of five 1-ms light pulses *(right)*. This Sst* neuron displays uEPSP with fast kinetics, little short-term dynamics and little summation, reminiscent of PV neurons.

(E) Input resistance and membrane time constant (Tau) are smaller in PV neurons compared to Sst neurons.

(F) AP threshold is similar for PV and Sst neurons. AP half-width is shorter in PV neurons compared to Sst neurons.

(G) Mean V_m is more hyperpolarized in PV neurons compared to Sst neurons. Spontaneous AP rate is higher in PV neurons compared to Sst neurons.

(H) The FFT of the V_m integrated over 1-5 Hz and cross-correlation between V_m and LFP at zero-time-lag are both larger in PV neurons compared to Sst neurons.

Data are represented as mean ± SD. Two-tail Wilcoxon rank-sum test assessed statistical significance. See also Table S1.

Figure S4

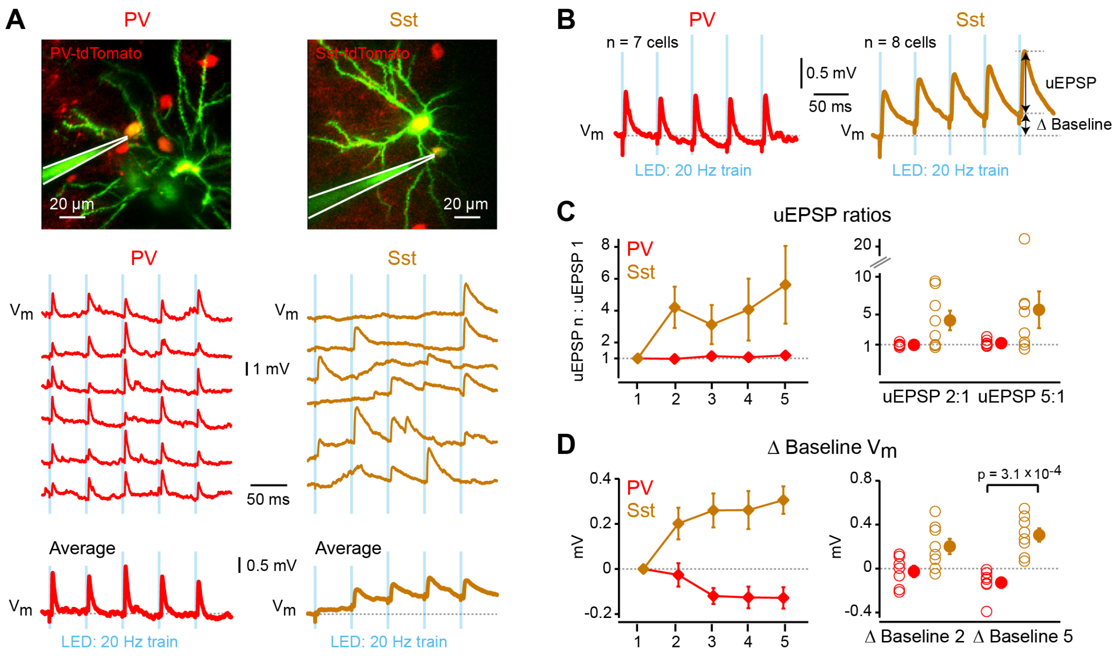

Figure S4. In vivo short-term synaptic dynamics for 20 Hz train of optogenetic stimuli, related to Figure 4.

(A) Example whole-cell recording of uEPSPs elicited in a PV (red) and Sst (brown) neuron during DOWN states by an optogenetic stimulus made of a 20 Hz train of five 1-ms light pulses. The neurons represented here are different than those in Figure 4. Single trial uEPSPs are shown above and average uEPSPs below. The in vivo two-photon images show the whole-cell recording pipette (Alexa 488 dye, green), the recorded tdTomato-expressing neuron (yellow) and part of the presynaptic eGFP- and ChR2-expressing neuron (green).

(B) Grand average uEPSPs for all connected PV and Sst neurons evoked by 20 Hz train of optogenetic stimuli during DOWN states.

(C) Population uEPSP amplitude ratios comparing the amplitude of each uEPSP in the train to the amplitude of the first uEPSP for PV and Sst neurons *(left)*. Individual neurons uEPSP amplitude ratios for uEPSP2 and uEPSP5 (*right*).

(D) Population difference in baseline V_m of each uEPSP in the train relative to the baseline V_m of the first uEPSP for PV and Sst neurons *(left)*. Differences across individual neurons in baseline V_m at onset of uEPSP2 and uEPSP5 *(right)*. uEPSPs summate in Sst neurons, but not in PV neurons.

Data are represented as mean ± SEM. Two-tail Wilcoxon rank-sum test assessed statistical significance.

Table S1

Properties	PV	Sst	Exc
Input resistance (MΩ)	**47 ± 22 (n = 21)**	**203 ± 45 (n = 43)**	**47 ± 13 (n = 27)**
Tau (ms)	**3.6 ± 2.5 (n = 21)**	**17.7 ± 6.4 (n = 45)**	**6.5 ± 1.6 (n = 27)**
Mean V_m (mV)	**-66.1 ± 6.0 (n = 52)**	**-59.9 ± 5.4 (n = 63)**	**-68.7 ± 5.6 (n = 54)**
AP rate (Hz)	**5.05 ± 4.05 (n = 38)**	**1.02 ± 1.56 (n = 37)**	**0.29 ± 0.64 (n = 54)**
AP threshold (mV)	**-36.7 ± 2.4 (n = 51)**	**-37.9 ± 3.0 (n = 33)**	**-38.1 ± 2.8 (n = 25)**
AP half-width (ms)	**0.32 ± 0.05 (n = 51)**	**0.62 ± 0.11 (n = 33)**	**1.05 ± 0.16 (n = 25)**
FFT area 1-5 Hz (mV)	**1.79 ± 0.50 (n = 35)**	**0.69 ± 0.41 (n = 35)**	**1.47 ± 0.51 (n = 49)**
V_m vs LFP cross-correlation	**0.74 ± 0.12 (n = 19)**	**0.42 ± 0.25 (n = 33)**	**0.71 ± 0.10 (n = 45)**

Table S1. Electrophysiological properties of parvalbumin-expressing GABAergic neurons (PV), somatostatin-expressing GABAergic neurons (Sst) and excitatory neurons (Exc), related to Figure 3.

Table S2

uEPSP property	PV	Sst	uEPSP property	PV	Sst
**Amplitude (mV)**	**n = 25**	**n = 17**	**Rise time (ms)**	**n = 25**	**n = 17**
mean ± SD	0.53 ± 0.39	0.50 ± 0.86	mean ± SD	0.68 ± 0.32	1.76 ± 1.40
median	0.39	0.21	median	0.61	1.31
range	0.03 – 1.40	0.02 – 3.48	range	0.33 – 1.85	0.58 – 5.89
**Failure rate (%)**	**n = 22**	**n = 16**	**Half-width (ms)**	**n = 24**	**n = 13**
mean ± SD	27.4 ± 15.5	68.0 ± 29.1	mean ± SD	4.0 ± 1.4	11.6 ± 6.7
median	31.7	80.2	median	4.3	9.1
range	0 – 51.0	4.5 – 90.3	range	1.2 – 6.6	3.9 – 23.1
**Coefficient of variation**	**n = 23**	**n = 16**	**Tau decay (ms)**	**n = 21**	**n = 9**
mean ± SD	0.33 ± 0.28	0.92 ± 0.53	mean ± SD	5.2 ± 3.0	16.0 ± 8.5
median	0.32	0.88	median	4.8	15.2
range	-0.31 – 0.94	0.26 – 1.92	range	1.3 – 12.7	6.0 – 32.9

Table S2. Properties of unitary EPSPs (uEPSP) recorded in parvalbumin-expressing GABAergic neurons (PV) and somatostatin-expressing GABAergic neurons (Sst), related to Figure 3.

Movie S1

Movie S1. Single-cell in vivo electroporation of DNA encoding eGFP and a fast variant of ChR2 together with Alexa 488 imaged using a two-photon microscope, related to Figure 1.

Positive pressure inside the electrode ejects green fluorescent dye (Alexa 488) and helps maintain the tip of the electrode clean. Unlabeled neurons in L2/3 are visualized as shadows. Upon electrode contact with the cell soma, a train of -12 V pulses each lasting 0.5 ms at a frequency of 50 Hz for 1 s delivers the DNA encoding eGFP and ChR2 together with Alexa 488 to the targeted neuron. Red fluorescence is from tdTomato-expressing neurons in the Sst-Cre x LSL-tdTomato mouse.

Supplemental Experimental Procedures

Animal preparation and surgery

All experiments were carried out with 4-8 week old female and male PV-IRES-Cre (Hippenmeyer et al., 2005) or Sst-IRES-Cre (Taniguchi et al. 2011) mice crossed with CAG-Lox-STOP-Lox-tdTomato (LSL-tdTomato) reporter mice (Madisen et al., 2010) in accordance with protocols approved by the Swiss Federal Veterinary Office. Mice were maintained under 1-2% isoflurane anesthesia while being implanted with a custom-made head-holder and a recording chamber. The location of the left C2 barrel column was functionally identified through intrinsic optical imaging under 0.5 - 1% isoflurane anesthesia (Lefort et al., 2009) and a small craniotomy (diameter 0.5 - 1 mm) was made taking care to leave the dura intact.

Single-cell electroporation

Electroporation of a single non-tdTomato neuron per PV-Cre x LSL-tdTomato or Sst-Cre x LSL-tdTomato mouse was carried out under 1% isoflurane anesthesia with slight modifications from a previously described protocol (Kitamura et al., 2008). In brief, a glass pipette with a resistance of 10-17 MΩ was filled with the same solution used for whole-cell recordings (see below) to which Alexa 488 dye (50-100 μM) (Invitrogen), pCAG-eGFP plasmid DNA (100 ng/μl) (Addgene plasmid 11150, kindly provided by Connie Cepko) (Matsuda and Cepko, 2004) and pCI-hSynapsin-ChR2(E123T/T159C) (200 ng/μl) (kindly provided by Thomas Oertner) (Berndt et al., 2011) were added. A two-photon microscope (Prairie Technologies) was used to visualize the pipette and the tdTomato-negative cell somas as dark shadows over a brighter background. The pipette was inserted in the brain through the intact dura and brought into close contact with the cell body of the target neuron and 50 pulses of negative voltage step (0.5 ms, –12 V) were delivered at 50 Hz using a pulse generator (Axoporator 800A, Molecular Devices). The craniotomy was then covered with a silicone elastomer (Kwik-Cast, WPI) and the mice were returned to their home cage for 24 hours before proceeding to electrophysiological recordings.

Electrophysiology

24 hours after electroporation, mice were re-anesthetized with 1-2% isoflurane and the dura was partially removed. Mice were placed again under the two-photon microscope and kept under 0.8-1.5% isoflurane anesthesia. The location of the single ChR2-expressing neuron was identified by the cortical blood vasculature pattern and its excitatory nature was confirmed by its overall morphology and the presence of numerous dendritic spines. Local field potential (LFP) was continuously recorded with a 2-4 MΩ glass pipette filled with Ringer solution containing 10-25 μM Alexa 594 dye and lowered at a depth of 150-250 μm from the pia and within 250 μm from the ChR2-expressing neuron. Two-photon targeted juxtacellular recording of the ChR2-expressing neuron was performed with 4-6 MΩ glass pipettes filled with the same solution as used for LFP recordings. Two-photon targeted whole-cell patch-clamp recordings were performed as previously described (Margrie et al. 2003, Gentet et al. 2010, Mateo et al. 2011). 5-7 MΩ glass pipettes were filled with a solution containing (in mM): 135 potassium gluconate, 4 KCl, 10 HEPES, 10 sodium phosphocreatine, 4 MgATP, 0.3 Na_3GTP (adjusted to pH 7.3 with KOH), to which 25-75 μM Alexa 488 dye and 3 mg/ml biocytin were added. Patch-clamp recordings were obtained in current-clamp mode and V_m was not corrected for liquid junction potentials. When current was injected to characterize intrinsic electrophysiological properties, series resistance subtraction was performed offline (see below). All recorded signals were amplified by a Multiclamp 700B amplifier (Axon Instruments), Bessel filtered at 10 kHz and digitized at 20 kHz by an ITC-18 (Instrutech Corporation) under the control of a custom program written in IgorPro (Wavemetrics).

Optogenetic stimulation

A collimated 470 nm superbright LED (Luxeon, Philips) was placed at the back of the two-photon objective to generate wide field stimulation. Optogenetic stimulus consisted of either a single square pulse of light of 1-ms duration and 10-70 mW/mm^2 intensity (mean ± SD: 32 ± 23 mW/mm^2), delivered with an interval of 1 s, or of a 20 or 50 Hz train of five 1-ms light pulses of similar intensity, delivered with a minimum interval of 5 s. A constant 470 nm background illumination made of an array of small LEDs (Everlight Electronics) was placed in front of the mouse during some recording sessions.

Histology and immunohistochemistry

After termination of the recordings, some mice were perfused with a 4% paraformaldehyde (PFA) solution, made by diluting a 32% PFA solution (EMS) in 0.1 M PBS. Brains were post-fixed for maximum 2 hours in the same solution, which was then replaced by a 0.1 M PBS solution. 50 µm thick coronal sections were cut using a semi-automated vibratome (VT1000S, Leica). Primary antibody against eGFP (rabbit, 1:5000, Abcam, Ab290) followed by secondary anti-rabbit antibody coupled to Alexa 488 (donkey, 1:200, Invitrogen) were used to enhance the eGFP signal of presynaptic neurons. Streptavidin coupled to Alexa 647 (1:2000, Invitrogen) was used to reveal biocytin filling of postsynaptic neurons. Images were obtained with a laser scanning confocal microscope (LSM 700, Zeiss) equipped with an oil-immersion 63x/1.4NA objective. Three-dimensional anatomical reconstructions were traced from confocal fluorescence image stacks using Neurolucida (MBF Bioscience).

Three 8-week old PV-Cre x LSL-tdTomato mice and three 8-week old Sst-Cre x LSL-tdTomato mice were used for single immunostains against parvalbumin (PV) or somatostatin (Sst), which were performed after a similar perfusion, post-fixation and slicing procedure as described above. Primary antibody against PV (rabbit, 1:1000, Swant, PV28) or primary antibody against Sst (rat, 1:200, Millipore, Mab354) were used. Secondary antibodies were goat anti-rabbit or goat anti-rat coupled to Alexa 647 (1:500, Invitrogen).

Three 7-week old Sst-Cre x LSL-tdTomato mice were used for double immunohistochemistry against both PV and Sst. Both primary and secondary antibodies to reveal Sst were the same as the one used for single immunohistochemistry. Primary antibody to reveal PV was also similar while secondary goat anti-rabbit coupled to Alexa 405 was used (1:200, Invitrogen). Images were obtained with the same laser scanning confocal microscope as above and either a 40x/1.3NA or a 63x/1.4NA oil-immersion objective. 405 nm, 555 nm and 639 nm solid state lasers were used to excite Alexa 405, tdTomato and Alexa 647 respectively. A 450 nm dichroic mirror followed by a 490 nm short pass filter were used to collect Alexa 405 fluorescence. A 500 nm dichroic mirror followed by a 505-600 nm band pass filter were used to collect tdTomato fluorescence. Alexa 647 fluorescence was collected using a 630 nm dichroic mirror followed by a 640 nm long pass filter. Colocalization of the markers with tdTomato fluorescence was assessed by careful inspection of the image stacks.

Data analysis

To assess state-specific optogenetic control of AP firing in ChR2-expressing excitatory neurons, periods of DOWN and UP state were identified through the simultaneously recorded LFP. In brief, the LFP was band pass filtered between 0.1 and 200 Hz and a sliding FFT (window size: 150 ms, overlap: 125 ms) was computed. Principal component analysis of the real part of the FFT followed by a Gaussian mixture model were used to extract and classify the twenty-five most relevant LFP frequency features of each window into three clusters, corresponding to UP, DOWN and transition states. An optogenetic stimulus was considered as occurring during DOWN state if the window before it and the second one after it were classified as belonging to the DOWN cluster. Similarly, a stimulus was considered as occurring during UP state if the window before it and the second one after it were classified as belonging to the UP cluster. APs were considered as optogenetically triggered if their peak happened within 20 ms of the end of the 1 ms light stimulus. AP latency was defined as the time elapsed between light stimulus onset and AP peak time. AP jitter was defined as the standard deviation of the AP latency. Each metric was separately computed for DOWN and UP states.

To investigate connectivity between pairs of neurons, initial analysis focused on optogenetic stimuli occurring during DOWN state only. DOWN and UP states were mostly identified using a double-threshold method applied directly on the V_m of individually recorded PV and Sst neurons. For PV neurons, the two thresholds were defined as the most hyperpolarized V_m value of the given recording sweep plus 5 mV, and plus 10 mV for threshold 1 and threshold 2 respectively. For Sst neurons, 3.5 mV and 5.5 mV were added to the most hyperpolarized V_m value of the given recording sweep to define threshold 1 and threshold 2. An optogenetic stimulus was considered as occurring during DOWN state if both the mean V_m averaged during the 10 ms preceding light onset and the mean V_m averaged during a time window ranging from 30 to 40 ms after light onset were smaller than threshold 1. Similarly, an optogenetic stimulus was considered as occurring during UP state if both average V_m values were larger than threshold 2. A subset of Sst neurons displayed minimal spontaneous V_m fluctuations, precluding the use of the double-threshold method. In such cases, DOWN and UP state identification was performed using the simultaneously recorded LFP as described above. A stimulus-triggered V_m average was computed for all optogenetic stimuli occurring during DOWN states (mean ± SD: 83 ± 43 stimuli; median: 82 stimuli) and compared with averaged spontaneous DOWN state V_m fluctuations. Recordings with less than 20 light stimuli occurring

during DOWN states were not considered for connectivity analysis. Neurons were considered to be synaptically connected if there was a clear difference between the average V_m traces with and without optogenetic stimulation, and that the timecourse was consistent with that of postsynaptic potentials. Specifically, we compared average V_m traces with and without optogenetic stimulation within a peak search-window, whose location and size was defined by the presynaptic ChR2-expressing neuron AP firing properties. It was set as the timing from "AP latency – AP jitter + 1 ms" to "AP latency + AP jitter + 3 ms" after light onset for PV neurons (peak-search window size: 3.1 ± 1.5 ms, mean ± SD) and that from "AP latency – AP jitter + 1 ms" to "AP latency + AP jitter + 5 ms" for Sst neurons (peak-search window size: 4.8 ± 0.5 ms, mean ± SD). Stimulus-triggered V_m averages of 2 PV neurons and 1 Sst neuron computed from less than 20 optogenetic stimuli were nonetheless considered for further uEPSP properties analysis, as they displayed clear synaptic connections.

Stimulus-triggered V_m average was used to quantify uEPSP amplitude and rise-time. uEPSP amplitude was calculated as the difference between the mean V_m averaged over a 0.5 ms window centered at the peak of the uEPSP and the mean baseline V_m averaged over a 1 ms window taken after the end of the optogenetic stimulus. uEPSP rise time corresponded to the time elapsed from 20% to 80% of the amplitude on the rising phase of the averaged uEPSP. uEPSP half-width and decay time constant (Tau decay) were extracted from a light stimulus-triggered V_m average made of a subset of trials elicited during DOWN states, which contained no major spontaneous V_m fluctuations from 10 to 20 ms after light stimulus onset. uEPSP half-width was calculated as the full width duration of the uEPSP at half of its maximum amplitude. Tau decay was determined by fitting a single exponential on the decaying phase of the averaged uEPSP, starting 1 ms after the peak and 2 ms after the peak for PV neurons and Sst neurons respectively. Synaptic transmission failure rate was estimated by calculating the fraction of all trials occurring during DOWN states where a clear uEPSP could not be detected within the same peak-search window as used to assess the presence or absence of a synaptic connection. To compute uEPSP amplitude coefficient of variation including failures, single trial uEPSP amplitude was measured as the difference between the mean V_m averaged over a 0.5 ms window centered at the peak of the uEPSP detected within the peak-search window (see above) and the mean baseline V_m averaged over a 1 ms window taken after the end of the optogenetic stimulus. Standard deviation of a similarly computed amplitude distribution for four DOWN state time points at which no light stimuli were applied was subtracted from the standard deviation of the obtained uEPSP amplitude distribution before dividing it by its mean in order to correct for spontaneous V_m

fluctuations occurring during DOWN states (Feldmeyer et al. 1999; Lefort et al. 2009).

For UP – DOWN state comparison of uEPSP amplitude, a light stimulus-triggered average of the V_m was obtained for UP and DOWN states separately. UP state trials where postsynaptic APs were present during a 30 ms (PV neurons) or a 50 ms (Sst neurons) time window starting 10 ms before light stimulus onset were omitted from the stimulus-triggered V_m average. Recordings with less than 20 optogenetic stimuli in either UP or DOWN states were not included in the analysis. UP state uEPSP amplitude was computed as described for DOWN state uEPSP amplitude. Peak-search window size was adjusted to match presynaptic ChR2-expressing neuron light-evoked AP firing properties during UP states.

To measure short-term synaptic dynamics, a stimulus-triggered average of the V_m was computed from all 20 Hz or 50 Hz optogenetic stimuli where the five light stimuli of the train occurred during DOWN states. Amplitudes of averaged uEPSPs during the stimulus train were analyzed as for single light pulses, except for the second to fifth uEPSPs in Sst neurons, where baseline V_m was extracted from the value taken by a single exponential fit of the decaying phase of the preceding uEPSP (fit start 2 ms after uEPSP peak) at the time of the current uEPSP peak.

Euclidean distance between the soma of the ChR2-expressing excitatory presynaptic neuron and the soma of the PV or Sst postsynaptic neuron was computed from two-photon image stacks acquired in vivo.

Electrophysiological properties of PV and Sst neurons were quantified as follows. Input resistance and membrane time constant (Tau) were measured by repeated current injections (-100 pA, 500 ms) immediately after establishing whole-cell configuration. Average V_m response was fitted offline with a double exponential from 0.4 ms to 50 ms after the onset of the current injection to determine and subtract the early fast component of the response, due to series resistance. Input resistance was calculated as the difference in the corrected mean V_m averaged over two 100 ms periods (one immediately before current injection and the other at the end of current injection) divided by the amount of injected current. Tau was determined by fitting the V_m with a single exponential from 1 ms to 60 ms after the onset of current injection. AP threshold was defined as the V_m at which the slope of rise of the voltage crossed 50 V/s (Kole and Stuart 2008). AP half-width was computed as the full width of the AP at half of its maximum amplitude measured from threshold to peak. Mean V_m and V_m FFT amplitude were computed across 20 s

sweeps of recording encompassing both UP and DOWN states. Spontaneous AP rate was computed across UP and DOWN states for the whole duration of the recording. To compute V_m *vs* LFP cross-correlation, V_m was offset by its average value and normalized by its standard deviation, and LFP was band pass filtered between 0.3 and 200 Hz.

Population data are represented as mean ± SD (except for Figures 4 and S4, where mean ± SEM is shown). Two-tail Wilcoxon rank-sum and signed-rank tests were used to compare two groups of unpaired and paired data respectively. χ^2 test was used to assess significant differences in connectivity rate. Spearman's ρ was used to quantify monotonic correlation between uEPSP amplitude and failure rate. Pearson's r was used to test for a linear relationship between connectivity rate and intersomatic distance. Data analysis was carried out in IgorPro (Wavemetrics) and Matlab (Mathworks) and statistical analysis was performed in Matlab.

Supplemental References

Feldmeyer, D., Egger, V., Lubke, J., and Sakmann, B. (1999). Reliable synaptic connections between pairs of excitatory layer 4 neurones within a single 'barrel' of developing rat somatosensory cortex. J. Physiol. *521*, 169-190.

Hippenmeyer, S., Vrieseling, E., Sigrist, M., Portmann, T., Laengle, C., Ladle, D.R., and Arber, S. (2005). A developmental switch in the response of DRG neurons to ETS transcription factor signaling. PLoS Biol. *3*, e159.

Kole, M.H., and Stuart, G.J. (2008). Is action potential threshold lowest in the axon? Nat. Neurosci. *11*, 1253-1255.

Madisen, L., Zwingman, T.A., Sunkin, S.M., Oh, S.W., Zariwala, H.A., Gu, H., Ng, L.L., Palmiter, R.D., Hawrylycz, M.J., Jones, A.R., et al. (2010). A robust and high-throughput Cre reporting and characterization system for the whole mouse brain. Nat. Neurosci. *13*, 133-140.

Margrie, T.W., Meyer, A.H., Caputi, A., Monyer, H., Hasan, M.T., Schaefer, A.T., Denk, W., and Brecht, M. (2003). Targeted whole-cell recordings in the mammalian brain in vivo. Neuron *39*, 911-918.

Matsuda, T., and Cepko, C.L. (2004). Electroporation and RNA interference in the rodent retina in vivo and in vitro. Proc. Natl. Acad. Sci. USA *101*, 16-22.

Taniguchi, H., He, M., Wu, P., Kim, S., Paik, R., Sugino, K., Kvitsiani, D., Fu, Y., Lu, J., Lin, Y., et al. (2011). A resource of Cre driver lines for genetic targeting of GABAergic neurons in cerebral cortex. Neuron *71*, 995-1013.
